# A *Vibrio*-based microbial platform for accelerated lignocellulosic sugar conversion

**DOI:** 10.1186/s13068-022-02157-3

**Published:** 2022-05-25

**Authors:** Sunghwa Woo, Hyun Gyu Lim, Yong Hee Han, Sungwoo Park, Myung Hyun Noh, Dongyeop Baek, Jo Hyun Moon, Sang Woo Seo, Gyoo Yeol Jung

**Affiliations:** 1 Department of Chemical Engineering, Pohang University of Science and Technology, 77 Cheongam-Ro, Nam-Gu, Pohang, 37673 Gyeongbuk Korea; 2 Interdisciplinary Program in Bioengineering, Seoul National University, 1 Gwanak-ro, Gwanak-gu, Seoul, 08826 Korea; 3 School of Chemical and Biological Engineering, Seoul National University, 1 Gwanak-Ro, Gwanak-Gu, Seoul, 08826 Korea; 4 Institute of Chemical Processes, Seoul National University, 1 Gwanak-ro, Gwanak-gu, Seoul, 08826 Korea; 5 Bio-MAX Institute, Seoul National University, 1 Gwanak-ro, Gwanak-gu, Seoul, 08826 Korea; 6 Institute of Engineering Research, Seoul National University, 1 Gwanak-ro, Gwanak-gu, Seoul, 08826 Korea; 7 School of Interdisciplinary Bioscience and Bioengineering, Pohang University of Science and Technology, 77 Cheongam-Ro, Nam-Gu, Pohang, 37673 Gyeongbuk Korea

**Keywords:** *Vibrio*, Lignocellulosic biomass, Xylose, Adaptive laboratory evolution, Carbon catabolite repression, Lactate

## Abstract

**Background:**

Owing to increasing concerns about climate change and the depletion of fossil fuels, the development of efficient microbial processes for biochemical production from lignocellulosic biomass has been a key issue. Because process efficiency is greatly affected by the inherent metabolic activities of host microorganisms, it is essential to utilize a microorganism that can rapidly convert biomass-derived sugars. Here, we report a novel *Vibrio*-based microbial platform that can rapidly and simultaneously consume three major lignocellulosic sugars (i.e., glucose, xylose, and arabinose) faster than any previously reported microorganisms.

**Results:**

The xylose isomerase pathway was constructed in *Vibrio* sp. dhg, which naturally displays high metabolic activities on glucose and arabinose but lacks xylose catabolism. Subsequent adaptive laboratory evolution significantly improved xylose catabolism of initial strain and led to unprecedently high growth and sugar uptake rate (0.67 h^−1^ and 2.15 g g_dry cell weight_^−1^ h^−1^, respectively). Furthermore, we achieved co-consumption of the three sugars by deletion of PtsG and introduction of GalP. We validated its superior performance and applicability by demonstrating efficient lactate production with high productivity (1.15 g/L/h) and titer (83 g/L).

**Conclusions:**

In this study, we developed a *Vibrio*-based microbial platform with rapid and simultaneous utilization of the three major sugars from lignocellulosic biomass by applying an integrated approach of rational and evolutionary engineering. We believe that the developed strain can be broadly utilized to accelerate the production of diverse biochemicals from lignocellulosic biomass.

**Supplementary Information:**

The online version contains supplementary material available at 10.1186/s13068-022-02157-3.

## Background

In recent decades, efficient conversion of lignocellulosic biomass (e.g., switchgrass, sorghum) into chemicals has been extensively studied owing to its high sugar content and abundance [1–4]. Various microorganisms such as *Escherichia coli* and *Saccharomyces cerevisiae* have been utilized and engineered to improve their catabolic activities for major sugars (i.e., glucose, xylose, and arabinose) [5–7]. These efforts have successfully demonstrated the potential of microbial processes for the sustainable production of diverse value-added chemicals.

Because process efficiencies are greatly affected by the innate metabolic activities of host microorganisms, it is essential to exploit a host that can efficiently and rapidly utilize sugars from lignocellulosic biomass (e.g., glucose, xylose, and arabinose) [8]. In this regard, *Vibrio* species have been recently suggested as a new powerful platform owing to their superior growth on various sugars over conventional host platforms [9–12]. In addition, its high tolerance to osmotic stress is expected to help improve biochemical production [13]. Indeed, a few pioneering studies have shown that a broad spectrum of biochemicals (e.g., ethanol, 2,3-butanediol, lycopene) can be produced from biomass sugars at high rates [9, 14–16] with the development of genetic toolboxes for controllable gene expression and genome editing [17]. These studies suggest that the use of *Vibrio* species would greatly expedite biochemical production from biomass.

To expand its application for lignocellulosic biomass conversion, several issues need to be addressed. A few *Vibrio* species (e.g., *Vibrio* sp. dhg and *Vibrio natriegens*) show no detectable growth or sugar consumption when grown on xylose, the second most abundant sugar in lignocellulose [10] (Additional file 1: Table S1), likely owing to their aquatic habitat [18]. Indeed, only 0.43% (61 out of 14,153) of *Vibrio* genomes deposited at the National Center for Biotechnology Information (NCBI) have complete sets of xylose catabolic genes. In contrast, more than 10% (338 out of 3,366) of *E. coli* strains have the genes (Additional file 1: Fig. S1). Furthermore, similar to many other microorganisms, the preferential utilization of sugars by carbon catabolite repression (CCR) [19] would lower the efficiency of bioprocesses [20]. Therefore, further studies to construct efficient catabolism of lignocellulose-derived sugars in *Vibrio* species are warranted to leverage its huge innate metabolic activity in bioprocessing.

**Fig. 1:**
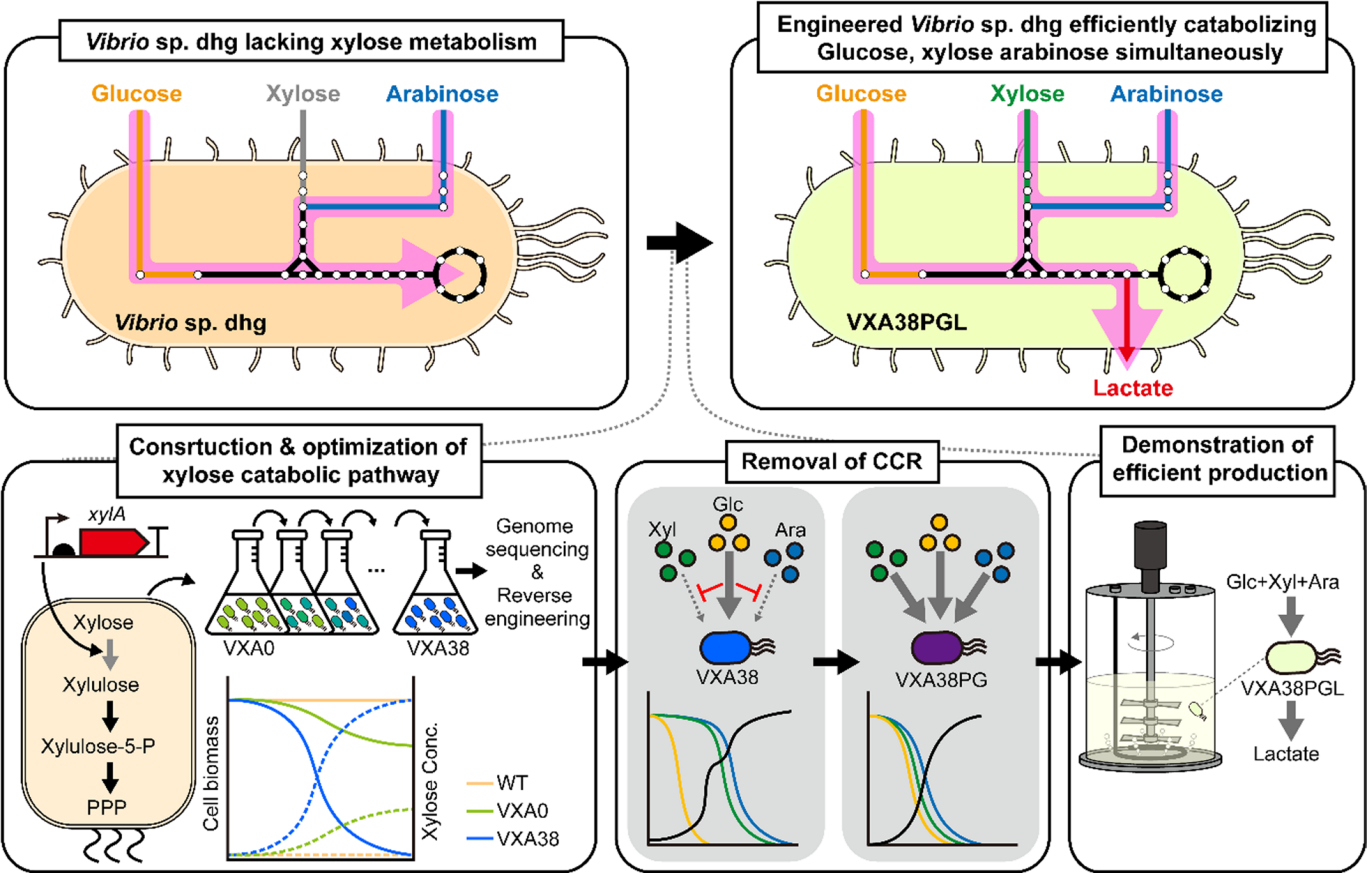
Generation of *Vibrio*-based microbial platform for efficient utilization of lignocellulosic sugars

In this study, we reported an engineered *Vibrio* sp. dhg that can rapidly and simultaneously utilize three major sugars (glucose, xylose, and arabinose) of lignocellulosic biomass (Fig. 1). First, based on the genome analysis of *Vibrio* sp. dhg, we heterologously expressed xylose isomerase from *E. coli* W, which was absent in *Vibrio* sp. dhg, to complete the xylose utilization pathway. This engineered strain was evolutionarily optimized by continuous growth in a xylose-supplemented minimal medium to achieve high xylose catabolic efficiency. Furthermore, we enabled the simultaneous utilization of glucose, xylose, and arabinose by deregulating its native glucose-induced CCR. Finally, we demonstrated the huge potential of the generated platform by achieving high lactate production (83 g/L in 72 h) with rapid co-consumption of sugars. Collectively, we believe that the developed strain will be widely utilized and greatly accelerate the production of diverse biochemicals from lignocellulosic biomass.

## Results

### Construction of the xylose isomerase pathway in *Vibrio* sp. dhg

**Fig. 2:**
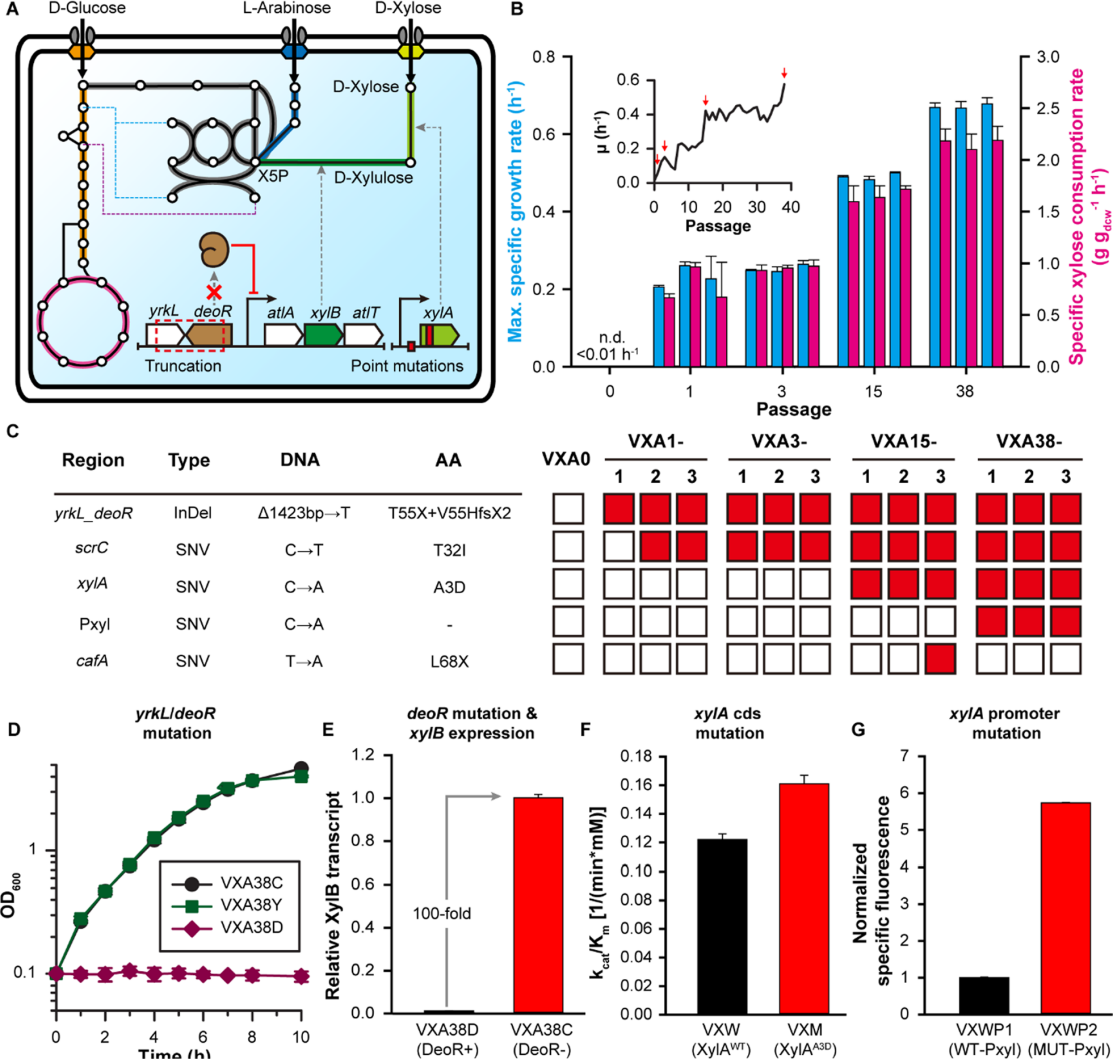
Improved xylose catabolism via ALE and elucidation of mutational mechanisms. **A** Predicted metabolic pathway of glucose, xylose, and arabinose in the VXA38 strain and genetic context of the region with major effective mutations. Key pathways are colored in yellow (glycolysis), gray (pentose phosphate pathway), pink (tricarboxylic acid cycle), blue (arabinose utilization pathway), and green (xylose isomerase pathway). Light and dark green indicate xylose isomerase (*xylA*) and xylulokinase (*xylB*), respectively. Light brown indicates the transcription factor for mannitol utilization family proteins (*deoR*), a putative repressor for *atlA*, *xylB*, and *atlT. yrkL, atlA*, and *atlT* encode NADH oxidoreductase, D-arabitol 4-dehydrogenase, and MFS superfamily transporters, respectively. Xylulose 5-phosphate is abbreviated as X5P. **B** Specific growth rates and xylose uptake rates of the isolates Blue indicates the maximum specific growth rate, and red indicates the specific xylose consumption rate. The subset graph indicates the specific growth rates in each flask during the ALE experiment. Red arrows indicate flasks selected for evolved clone isolation. **C** Mutation analysis of the starting and evolved strains. The red boxes indicate the presence of mutations. V55Hfs2X means that the 55^th^ amino acid was changed from valine to histidine (V55H) and the stop codon was generated after 2 amino acids (57^th^) owing to the frameshift (fs2X). X indicates the generation of a stop codon. **D** Cell growth (optical density at 600 nm, OD_600_) over time with xylose minimal medium. Symbols: black circle, VXA38C (VXA38-1 with empty vector); green square, VXA38Y (VXA38-1 with additional *yrkL* expression); purple diamond, VXA38D (VXA38-1 with additional *deoR* expression). **E** Relative amounts of *xylB* transcripts in the VXA38C and VXA38D strains. **F** Catalytic efficiencies (k_cat_/K_m_ in min^−1^ mM^−1^) of wild-type (VXW) and mutant (VXM) xylose isomerase **G** Normalized specific fluorescence of strains expressing the *xylA-sgfp* fused protein under the original P_J23100_ (VXPW) and mutant promoter (VXPM)

We analyzed the genome of *Vibrio* sp. dhg to identify its endogenous pathways for sugar catabolism and to determine the genes required for xylose utilization. To this end, we queried the names of essential enzymes in each sugar catabolic pathway from the annotated reference genome of *Vibrio* sp. dhg [9]. If there was no matched enzyme name, we queried the amino acid sequence of an enzyme from a representative microorganism using Protein BLAST [21]. Genomic analysis revealed that *Vibrio* sp. dhg can catabolize glucose and arabinose via the Embden–Meyerhof–Parnas (EMP) pathway and pentose phosphate pathway (PPP) (Fig. 2A, Additional file 1: Table S2). Still, it does not have any complete gene sets of four known xylose utilization pathways (i.e., the isomerase pathway, oxidoreductase pathway, Weimberg pathway, and Dahms pathway, Additional file 1: Fig. S2). Considering that the xylose isomerase pathway provides the highest carbon yield and energy generation [22] (Additional file 1: Tables S3, 4), we decided to construct the xylose isomerase pathway in *Vibrio* sp. dhg.

To achieve this goal, we expressed heterologous *xylA* from *E. coli* W in *Vibrio* sp. dhg, which was the only required gene to construct the xylose isomerase pathway; all other genes including *xylB* encoding xylulokinase, another key enzyme for xylose isomerase pathway, were identified in *Vibrio* sp. dhg (Additional file 1: Table S2). To ensure the stable and constitutive expression of *xylA*, we used a synthetic promoter (VP13, equivalent to P_J23100_) and an optimized 5'-UTR generated by UTR Designer [23]. Furthermore, the expression cassette was integrated into the chromosome by replacing *dns* encoding an extracellular nuclease; this gene was known to be non-essential for cell viability and its deletion enhances transformation efficiency [24]. Notably, the resulting VXA0 strain showed growth on xylose as the sole carbon source by successfully activating the xylose isomerase pathway. However, the strain showed a long lag phase (up to 4 days) before its growth and a low growth rate (0.01 h^−1^), indicating a necessity for further optimization.

### Evolutionary optimization of xylose catabolism in *Vibrio* sp. dhg

We applied an adaptive laboratory evolution (ALE) strategy [25–27] to further improve xylose utilization (Fig. 2B). Given that the VXA0 strain grew on xylose as a sole carbon source, we grew and iteratively passaged this strain in a xylose-supplemented minimal medium (see Methods for detail). Surprisingly, the population in the second flask displayed no lag phase time and a significantly increased growth rate (0.1 h^−1^). Thereafter, the growth rates of the populations gradually increased over time (Fig. 2B). Within 2 months, a dramatically improved growth rate of 0.58 h^−1^ was achieved in the 38^th^ flask. This observation implied the generation and accumulation of novel mutations augmenting xylose catabolism. The evolved population underwent 164 generations, equivalent to 1.4 × 10^12^ cumulative cell divisions (CCD).

For a detailed characterization, we isolated evolved strains from multiple timepoints. We evaluated their maximum specific growth rates and specific sugar consumption rates in the xylose minimal medium (Fig. 2B). Specifically, three clones were isolated from four timepoints (a total of 12 clones): three intermediate timepoints (the 1^st^, 3^rd^, and 15^th^ flasks), where clear jumps in growth rates were observed, and the endpoint of the ALE experiment (the 38^th^ flask). The growth rates of the isolated clones generally showed a similar trend to the population growth rates; higher growth rates were observed with clones isolated from later flasks. In addition to increases in growth rates, xylose uptake rates were greatly improved. Resultantly, the three endpoint isolates showed the growth rates and specific xylose uptake rates of 0.67 h^−1^ and 2.15 g g_dcw_^−1^ h^−1^, respectively, as a maximum. It should be noted that these values were superior to those of any other reported microbial platforms [5, 22, 28–31] that consume xylose (Additional file 1: Table S5).

### Identification and validation of beneficial mutations improving xylose utilization

Whole-genome sequencing was performed for the VXA0 strain and the evolved isolates to identify mutations that improved xylose utilization (Fig. 2C). We identified five mutations in four regions in the genomes of the evolved isolates by the comparison with the genome of the starting strain (Additional file 1: Table S6, 7): (i) an deletion mutation substituting 1423 bases (Chr2; 1,846,276–1,847,699) into T of two neighboring genes (*yrkL* encoding an NADH oxidoreductase and *deoR* encoding a transcription factor for mannitol utilization family proteins), (ii) a single nucleotide variation (SNV) mutation in *scrC* encoding bifunctional diguanylate cyclase/phosphodiesterase (Chr2; 1,409,023, C to T resulting T32I), (iii and iv) SNV mutations in the promoter (Chr1; 2,768,285) and coding sequence (Chr1; 2,768,325, C to A resulting A3D) of the *xylA* gene, and (v) a SNV mutation in *cafA* encoding a cytoplasmic axial filament protein (Chr1; 2,875,221, T to A resulting early termination). Among these mutations, three mutations (i, iii, and iv) appear to substantially improve the maximum specific growth rate on xylose. While the mutation in *scrC* did not affect growth during the exponential phase, it significantly reduced the lag time (Additional file 1: Fig. S3). It is likely that the *cafA* mutation does not affect xylose catabolism, given that no significant difference was observed between the VXA15-1 and VXA15-3 strains (Additional file 1: Fig. S3) and the mutation did not persist.

To understand how xylose catabolism was improved during exponential growth, we characterized the effects of the three mutations (i, iii, and iv). Initially, the effect of the mutation in *yrkL* and *deoR* was studied, since it first occurred, and an operon, divergently expressed next to *deoR,* contained a putative xylulokinase gene (52% amino acid identity of XylB from *E. coli*, Fig. 2A and Additional file 1: Fig. S4). This operon additionally contains *atlA* and *atlT* which encode D-arabitol 4-dehydrogenase and MFS superfamily transporter, respectively; Xylulokinase has often been found from arabitol utilization operons in many microorganisms [32–34], suggesting the importance of the putative *xylB* gene in the xylose metabolism in *Vibrio* sp. dhg. Initially, we individually expressed the intact *yrkL* and *deoR* genes in the endpoint isolate, VXA38-1, using a plasmid. The resulting VXA38Y (VXA38-1 with *yrkL* expression), VXA38D (VXA38-1 with *deoR* expression), and VXA38C (VXA38-1 with an empty plasmid as a control) strains were cultivated in xylose minimal medium (Fig. 2D). Notably, DeoR complementation completely impaired growth on xylose, whereas YrkL expression did not affect growth. Next, we analyzed the expression level of *xylB* upon DeoR expression; *xylB* was barely expressed (up to 100-fold, Fig. 2E), confirming the importance of this *xylB* gene in xylose metabolism. Collectively, these observations showed that the insufficient activity of xylulokinase was one of the rate-limiting steps in xylose metabolism, and higher growth was achieved by the truncation of DeoR, which upregulated *xylB* expression.

Next, we investigated the roles of these two mutations in the *xylA* coding sequence and its promoter region. Since the coding sequence mutation is non-synonymous, it was expected that the activity of XylA would be affected. Thus, we compared the specific activities (k_cat_/K_m_) of the purified mutant and wild-type XylA. Notably, it was found that the A3D mutation resulted in a 1.3-fold higher catalytic efficiency (k_cat_/K_m_) (Fig. 2F and Additional file 1: Fig. S5). Given that the mutated residue is located at the N-terminus, far from known active sites of similar XylA in other microorganisms [35, 36], this mutation likely affects the assembled structure of its homotetramer; a further detailed study is warranted. Nevertheless, this analysis confirmed that the higher activity of XylA enhanced xylose catabolism.

Finally, we investigated the effect of mutations on the expression levels of *xylA*. Since they are located in either the -10 box of P_J23100_ or the proximal region to the start codon, it was likely that *xylA* expression was affected. In particular, the mutated promoter sequence became more similar to the consensus sequence of bacterial promoters [37], suggesting that the mutation potentially led to a higher expression of *xylA*. For validation, we quantified the amount of XylA by generating a fusion protein of the wild-type or mutant XylA with a green fluorescent protein (sGFP) expressed under the wild-type and mutant promoters (Fig. 2G). It was found that the amount of the fusion protein was sixfold higher with the mutant promoter compared to that with the wild-type promoter, whereas the mutation in the coding sequence did not affect the expression level (Additional file 1: Fig. S6). Collectively, the low activity of XylA was a bottleneck for xylose catabolism, and its expression cassette was mutated to increase its transcription level and specific enzyme activity.

### Enabling simultaneous utilization of glucose, xylose, and arabinose

**Fig. 3:**
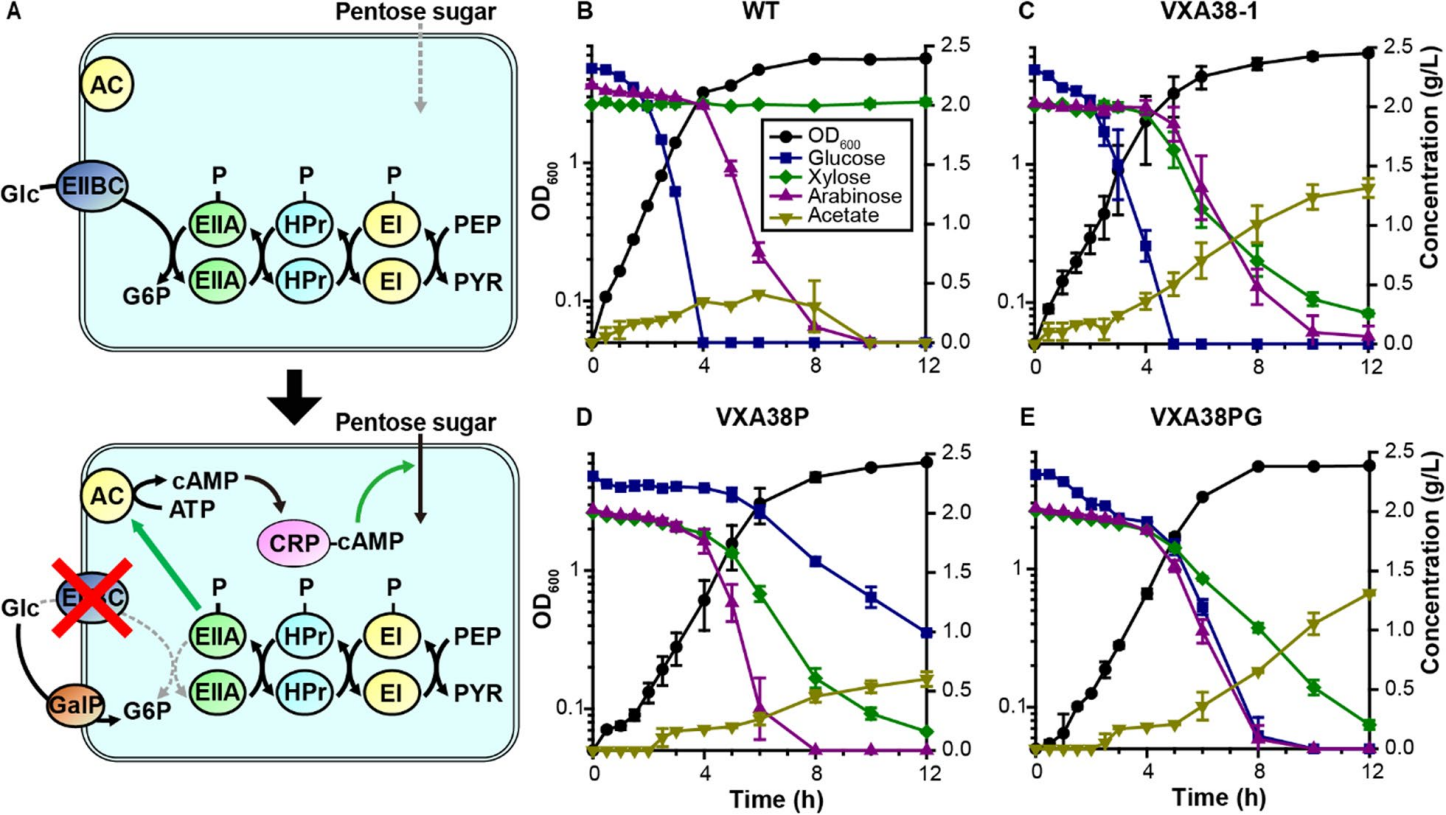
Engineering for simultaneous utilization of lignocellulose-derived sugars. **A** Altered PTS to enable co-consumption of glucose, xylose, and arabinose. In the wild type, CRP cannot activate genes for arabinose and xylose utilization. Inactivation of PtsG increases the concentration of phosphorylated EIIA, which activates AC and increases cAMP concentration. CRP activated by cAMP promotes the transcription of genes related to the catabolism of xylose and arabinose. Glucose can be transported through GalP instead of EIIBC. Abbreviations: Glc, glucose; Xyl, xylose; Ara, arabinose; G6P, glucose-6-phosphate; PEP, phosphoenolpyruvate; PYR, pyruvate; EI, enzyme I; HPr, histidine protein; EIIA, enzyme II A component; EIIBC, enzyme II BC component; AC, adenylate cyclase; ATP, adenosine triphosphate; cAMP, cyclic adenosine monophosphate; CRP, cAMP receptor protein; GalP, galactose–proton symporter. Growth profiles of **B** wild-type *Vibrio* sp. dhg, **C** VXA38-1, **D** VXA38P, and **E** VXA38PG strains in minimal medium containing 2 g/L of glucose, xylose, and arabinose. The left and right *y*-axes indicate cell biomass (OD_600_) and sugar concentration (g/L), respectively, and the *x*-axis indicates time (h). Symbols: black circles, OD_600_; blue squares, glucose; green diamonds, xylose; purple triangles, arabinose; yellow inverted triangle, acetate

We further engineered the VXA38-1 strain to simultaneously utilize the major sugars (glucose, xylose, and arabinose) obtained from lignocellulose (Fig. 3A). Cultivation of the wild-type and VXA38-1 strains with a mixture of the three sugars (Fig. 3B, C) showed that the xylose and arabinose catabolism is repressed in the presence of glucose, similar to many other bacteria [38–40]; after glucose was depleted, xylose and arabinose were consumed simultaneously. In many bacteria, including *Vibrio* species [41–44], it is known that glucose is preferentially utilized by suppressed gene expression of the non-favored sugar utilization pathway by the cAMP receptor protein (CRP) (Fig. 3A). Given that the activity of adenylate cyclase (AC), which controls intracellular cAMP levels, is regulated by the phosphotransferase system (PTS) [40, 45], it was shown that altered PTS by the deletion of PtsG (a key enzyme consisting of PTS, EIIBC) enabled co-consumption of multiple sugars in *E. coli*, *Klebsiella oxytoc*a*, Enterobacter aerogenes*, and *Corynebacterium glutamicum* [38–40]. Similarly, we also tested whether the deletion of *ptsG* could enable the co-consumption of sugars. Consistent with previous studies, this strain successfully co-utilized the three sugars (Fig. 3D). However, the glucose utilization and byproduct (i.e., acetate) formation were severely reduced, indicating the necessity for further optimization [41].

To restore glucose transportation, we additionally expressed an alternative non-PTS galactose symporter, GalP, from *E. coli* in VXA38P (Fig. 3A). Although its primary substrate is galactose, it can also uptake glucose without affecting the cAMP level [45, 46]. Surprisingly, the resulting VXA38PG strain showed a substantial increase in glucose uptake without affecting the simultaneous utilization of xylose and arabinose (Fig. 3E). Moreover, the total sugar consumption rate was significantly increased in VXA38PG (2.01 g g_dcw_^−1^ h^−1^) strain compared to the VXA38P strain (1.69 g g_dcw_^−1^ h^−1^). The recovered acetate production also confirmed its potential to serve as a production host. Considering the rapid and simultaneous utilization of all major sugars in lignocellulose, the resulting strain was further engineered for chemical production.

### Efficient lactate production from lignocellulose-derived sugars

**Fig. 4:**
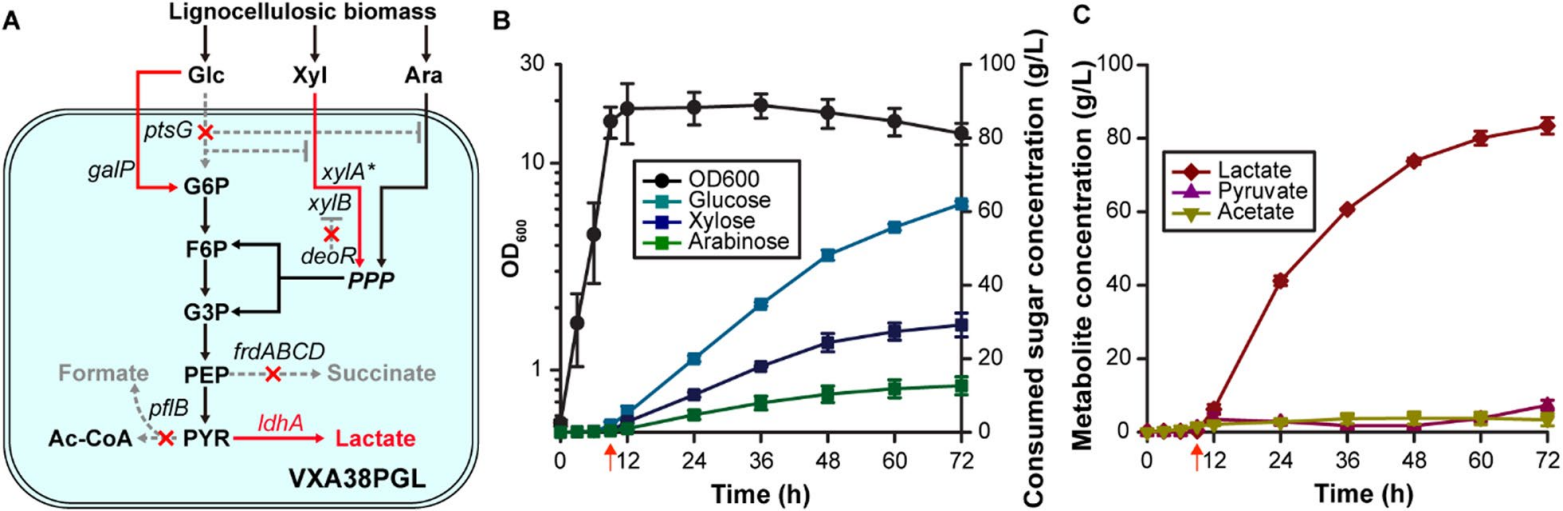
Lactate production using the VXA38PGL. **A** Schematic lactate production pathway in VXA38PGL **B** Growth and sugar consumption profiles of the VXAPGL strain. The left and right *y*-axes indicate the cell biomass (OD_600_) and consumed sugar concentration (g/L), respectively. The *x*-axis indicates the time (h). **C** Lactate and byproduct production profile of the VXAPGL strain. The *x*- and *y-*axe*s* indicate time (h) and product titer (g/L), respectively. Red arrows indicate the timepoint when the oxygen supply was stopped, and nitrogen gas was supplied. Symbols: black circles, OD_600_; blue squares, glucose; dark blue squares, xylose; green squares, arabinose; brown diamonds, lactate; purple triangles, pyruvate; dark yellow inverted triangles, acetate

We applied the developed VXA38PG strain, which can rapidly and simultaneously utilize the three lignocellulosic sugars for biochemical production. As a model compound, we chose lactate, which has various industrial applications, such as an acidulant, a preservative, and a monomer for biodegradable plastics [47] (Fig. 4A). To efficiently produce lactate, byproduct-producing pathways were blocked by deleting fumarate reductase (*frdABCD*) and pyruvate-formate lyase (*pflB*) in the genome. Furthermore, endogenous lactate dehydrogenase (*ldhA*) was overexpressed in the plasmid.

We cultivated the engineered strain, VXA38PGL, in a medium (Fig. 4B) supplemented with a mixture of the three sugars (i.e., glucose, xylose, and arabinose) at a ratio of 6:3:1, mimicking the contents of these sugars in lignocellulose [48, 49]. Mimetic sugar (40 g/L) was periodically supplemented with a 12 h interval. As a result, 83 g/L of lactate was produced over 72 h, which corresponds to a productivity of 1.15 g/L/h. Notably, byproduct formation was minimized, resulting in a high yield (0.80 g/g, 133 g of lactate from 166 g of the total sugars), equivalent to 80% of the theoretical maximum yield (Fig. 4C). The titer was the highest, and the yield was comparable with those of other similar studies (Additional file 1: Table S8). Moreover, 1.4-fold higher productivity was achieved compared with that of *E. coli* in a medium with an identical sugar composition by leveraging the high metabolic efficiency of *Vibrio* sp. dhg. These results collectively support the potential of the strain as a platform for the lignocellulose-based fermentation process due to its high performance.

## Discussion

This study supports the power of the ALE strategy for generating platform strains for strain. Although rational engineering approaches have been widely applied to engineer microorganisms, they are often limited due to insufficient comprehension of the complex and multilayered network in microorganisms. In this regard, the ALE strategy can efficiently complement the limitations of rational engineering, particularly for growth-associated phenotypes [50, 51]. Rate-limiting steps in a given microorganism can be identified and autonomously optimized by natural selection. Indeed, it was found that the low activities of XylA and XylB were bottlenecks, and they were evolutionarily optimized by ALE.

Although the effect of ScrC mutation was not studied in detail, further studies are needed to elucidate its role in *Vibrio* species. It has been reported that ScrC in *Vibrio* species controls the intracellular level of cyclic di-GMP (c-di-GMP) by converting two molecules of GTP into c-di-GMP followed by c-di-GMP into pGpG via its diguanylate cyclase activity and phosphodiesterase activity [52–54]. C-di-GMP is known to be involved in bacterial global stress responses by regulating the expression of genes related to motility, biofilm formation, and virulence factors [55, 56]. Although mutations in a global stress response mechanism have been commonly observed in recent ALE studies [57–59], additional studies are needed to understand the clear mutational mechanism for removing lag in xylose conditions. Potentially, c-di-GMP might directly affect the expression of genes related to xylose catabolism or indirectly affect catabolism via reduced biofilm formation, which is important for overall planktonic cell growth and sugar utilization [60, 61].

To apply the developed platform for actual lignocellulose conversion, further fermentation studies with biomass hydrolysates are warranted. Potentially, its performance could change with actual hydrolysates, since they are known to contain diverse growth-inhibiting compounds, such as furfural, 5-hydroxymethylfurfural, and levulinic acid, which originated from the degradation of sugars during acid/heat treatments [62, 63]. This issue could be overcome by optimizing pretreatment to minimize the formation of toxic compounds and maximize sugar yields, which have been actively investigated [64–66]. Alternatively, the developed strain can be subjected to another round of ALE to tolerize it against toxic compounds.

Finally, *Vibrio* species have high potential as a novel microbial chassis for the bio-based industry. The high metabolic efficiency of *Vibrio* species can improve the productivity of any target compounds [9, 10, 17], which is greatly helpful in increasing the economic feasibility of microorganism-based biochemical production processes. Moreover, its remarkable growth shortens biological experiments, making it a suitable chassis for research purposes, such as molecular biology, evolutionary biology, and protein engineering [11, 12, 67]. Therefore, further studies on the deployment of *Vibrio* species as microbial platforms are highly promising.

## Conclusions

In this study, we developed a *Vibrio*-based microbial platform with rapid and simultaneous utilization of the three major sugars from lignocellulosic biomass by applying an integrated approach of rational and evolutionary engineering. We constructed a xylose isomerase pathway by heterologous chromosomal expression of the xylose isomerase gene based on genomic analysis. Furthermore, we obtained an efficient strain displaying a growth rate of 0.67 h^−1^ and a sugar uptake rate of 2.15 g g_dcw_^−1^ h^−1^ on xylose, via ALE. It was confirmed that the evolved strain catabolized xylose at a faster rate than any reported microorganism (Additional file 1: Table S5). Subsequent mutation analysis and reverse engineering revealed that the improved phenotype was achieved by autonomous optimization in a multi-level process for catabolizing xylose. We then achieved simultaneous utilization of glucose, xylose, and arabinose by removing CCR in the strain and demonstrated efficient lactate production with remarkable productivity.

## Methods

### Microbes and culture media

*Escherichia coli* strains were cultured in LB medium with appropriate antibiotics at 37 °C. *Vibrio* sp. dhg and its derivative strains were cultivated in the buffered minimal medium (5 g/L (NH_4_)_2_SO_4_, 30 g/L NaCl, 10.7 g/L K_2_HPO_4_, 5.2 g/L KH_2_PO_4_, 0.5 g/L MgSO_4_·7H_2_O, and 2 mL L^−1^ trace metal solution (ATCC MD-TMS), supplemented with various concentrations of carbon sources and yeast extract) and LBv2 medium (10 g/L tryptone, 5 g/L yeast extract, 21.92 g/L NaCl, 0.3 g/L KCl, and 2.2 g/L MgCl_2_) with appropriate antibiotics at 37 °C [9]. Agar plates were prepared by including 15 g/L of agar into the media. The pH of all media and a buffer was adjusted to 7.

### Culture conditions

For routine cell cultures at the flask scale, colonies were picked from LB or LBv2 medium agar plates and inoculated in 3 mL of M9 or buffered medium supplemented with 4 g/L of sugar (glucose, xylose, and arabinose) contained in 15 mL test tubes. After overnight incubation, the culture was re-inoculated into a fresh medium at an OD_600_ (optical density at 600 nm) of 0.05. When the OD_600_ reached 1.0, cells were transferred into a 350 mL Erlenmeyer flask containing 25 mL of the medium at an OD_600_ of 0.05. Cultures were conducted in a rotary shaker (Hanil Scientific, Gimpo, Korea) at 37 °C and 200 rpm. Appropriate antibiotics were supplemented. All cell cultures were conducted in triplicates. OD_600_ was measured using a UV-1700 spectrophotometer (Shimadzu, Kyoto, Japan). OD_600_ of 1.0 corresponds to 0.31 g_dcw_ /L and 0.27 g_dcw_ /L for *E. coli* and *Vibrio* sp. dhg, respectively. The maximum specific growth rate (μ, h^−1^) was calculated by linear regression of ln(OD_600_) and time (h) during the exponential growth phase. Maximum specific sugar uptake rates (g g_dcw_^−1^ L^−1^) were calculated by dividing the maximum specific growth rates by the biomass yields. The cumulative cell division number was calculated by summation of division events calculated from the initial cell number and the total number of generations in a flask [68]. An OD_600_ of 1 was regarded as an 8 × 10^8^ cell number [69].

For a bioreactor scale culture with a mimetic sugar medium, colonies were picked from LBv2 medium agar plates, inoculated in 3 mL of LBv2, and cultured overnight. Subsequently, the culture was refreshed by inoculating into several flasks containing 50 mL of LBv2 supplemented with 20 g/L of a sugar mixture (glucose:xylose:arabinose = 6:3:1) in 350 mL Erlenmeyer flasks at an OD_600_ of 0.1. When the OD_600_ reached 1–2, cells were harvested and transferred into a 7 L bioreactor (Biotron Limited, State of New South Wales, Australia) containing 1 L of the medium at an OD_600_ of 0.5. Cultures were incubated at 37 °C and 800 rpm. The pH was maintained at 6.5–7.0, using a pH controller (Biotron Limited). During the aerobic culture phase, oxygen gas was supplemented at 4 L/min until an OD_600_ of 12–15 was achieved. Thereafter, the cells were grown anaerobically by providing nitrogen gas at a rate of 2 L/min. The sugar feeding stock solution (300 g/L glucose, 150 g/L xylose, 50 g/L arabinose, 5 g/L yeast extract, 10 g/L tryptone, and 10 μg/mL chloramphenicol) was intermittently added when the total sugar concentration was below 10 g/L; each feeding increases the total sugar concentration by approximately 20 g/L. At least three identical cultures were independently performed to confirm the reproducibility.

### Construction of strains and plasmids

Bacterial strains and plasmids are listed in Additional file 1: Table S9 and the primers, synthesized by Cosmogenetech (Seoul, Korea), are listed in Additional file 1: Table S10. Detailed plasmid construction methods are organized in Additional file 1: Table S11. Plasmid and genomic DNA were prepared using a GeneAll^R^ Exprep™ Plasmid SV kit and Exgene™ Cell SV kit (GeneAll, Seoul, Korea), respectively. For purification of fragmented DNA, we used an Expin™ Gel SV kit (GeneAll, Seoul, Korea). For cloning, Q5 polymerase, a NEBuilder^R^ HiFi DNA Assembly Cloning Kit, restriction enzymes, and Quick Ligation™ kit were purchased from New England Biolabs (Ipswich, MA, United States). For routine colony PCR, EmeraldAmp^R^ GT PCR Master Mix was used (Takara Bio Inc., Kusatsu, Japan).

The recombination was performed as the previous study [9] using pCDF_xylA_ins, pCDF_ptsG_del, pCDF_frdABCD_del, pCDF_pflB_del plasmids. dsDNA fragment for the integration of *xylA* gene, which contains homology adjacent to *dns* gene, FRT_cat_FRT, and *xylA* gene overexpression cassette was amplified using xylA_1K_F and xylA_1K_R primer with pCDF_xylA_ins as a template. dsDNA fragments for deletion of *ptsG*, *frdABCD*, and *pflB* gene, which contain homology adjacent to the target gene and FRT_cat_FRT was amplified using [gene name]_1K_F and [gene name] _1K_R primers with pCDF_[gene name]_del as a template. Recombination was confirmed by PCR with [gene name]_ch_F/R primers and sanger sequencing.

*xylA* gene was integrated into *Vibrio* sp. dhg wild-type strain to construct VXA0 strain. After ALE, VXA38C, VXA38Y, and VXA38D strains were constructed by transforming pACYC_Duet, pACYC_yrkL, and pACYC_deoR plasmids into VXA38 strain, respectively. VXW, VXM, VXWP1, VXMP1, VXWP2, and VXMP2 strains were constructed by transforming pACYC_xylAWT_Histag, pACYC_xylAMUT_Histag, pACYC_PWT_xylAWT_sgfp, pACYC_PWT_xylAMUT_sgfp, pACYC_PMUT_xylAWT_sgfp, and pACYC_PMUT_xylAMUT_sgfp plasmids into wild-type *Vibrio* sp. dhg strain, respectively. VXA38P strain was constructed by knockout of *ptsG* gene in VXA38 strain and VXA38PG strain was constructed by transformation of pACYC_galP plasmid into VXA38P strain. VXA38PGL strain was constructed by knockout of *frdABCD* and *pflB* gene and transformation of pACYC_galP_ldhA plasmid in VXA38P strain.

### Adaptive laboratory evolution in xylose sole carbon source condition

ALE was performed at the flask scale by growing cells in a xylose-supplemented minimal medium. A single colony of the VXA0 strain from an LBv2 agar plate was inoculated into 3 mL of LBv2 medium. After overnight incubation, the culture was washed twice with a minimal buffered medium without any carbon sources. Then, cells were inoculated in 25 mL of the medium supplemented with 4 g/L xylose in 350 mL Erlenmeyer flasks at an OD_600_ of 0.1. When the OD_600_ was higher than 2, the cultures were passaged to the next flask at an OD_600_ of 0.1. The ALE was completed once a growth rate of 0.6 h^−1^ was achieved.

### Whole-genome sequencing and mutation identification

Genomic DNA was extracted from cells grown in LB medium using a GeneAll Exgene™ Cell SV kit (GeneAll Biotechnology, Seoul, Korea). Pair-end libraries were prepared using the KAPA HyperPlus Kit (KAPA Biosystems, Wilmington, MA, USA). Raw reads were obtained using a MiniSeq 300-cycle Mid-Output kit on the MiniSeq system (Illumina, San Diego, CA, USA). Mutations were identified using the Breseq analysis software (version 0.33.2) [70]. A new reference genome of the VXA0 strain was generated based on *Vibrio* sp. dhg (NCBI accession number: CP028943.1, CP028944.1, and CP028945.1) to include the introduced *xylA* and to identify mutations in this strain. All genomes were sequenced with at least 25 × sequencing coverage. Raw sequencing files were deposited at SRA (Bioproject number: PRJNA720008). All discovered mutations were validated by Sanger sequencing.

### Quantification of the *xylB* transcript levels

*XylB* transcripts were measured by quantitative PCR (qPCR) using *rpoA* as a reference. The total RNA of the VXA38D and VXA38C strains in the mid-log phase were extracted using a Ribospin II kit (GeneAll, Seoul, Korea). Complementary DNA of *rpoA* and *xylB* mRNA for each sample was synthesized using M-MLV Reverse Transcriptase (Elpis-Biotech, Daejeon, Korea) and rpoA_RT_R and xylB_RT_R primers. qPCR was performed in technical triplicates using TOPreal™ qPCR 2 × PreMIX (SYBR Green with high ROX) (Enzynomics, Daejeon, Korea) and rpoA_RT_F/R and xylB_RT_F/R primer sets, designed to amplify 200 bp regions of each gene (Additional file 1: Table S10). A StepOnePlus Real-time PCR system (Applied Biosystems, Foster City, CA, USA) was used for amplification and signal detection. To determine the relative transcript amount, the comparative CT method (2^−ΔΔCT^) was utilized [71].

### Measurement of the specific activity of xylose isomerase

Specific activities of the purified xylose isomerases were determined using an enzymatic assay. Cells harboring 6His-tagged XylA were grown in LBv2 medium and lysed at an OD_600_ of 0.8–1, by mixing with BugBuster Master Mix (Merck, Darmstadt, Germany). XylA was purified using a MagListo™ His-tagged protein purification kit (Bioneer, Daejeon, Korea) and concentrated using an Amicon Ultra Centrifugal Filter (Merck, Darmstadt, Germany). The purified protein amount was quantified by following the Bradford assay with bovine serum albumin (BSA) as a reference [72]. The concentration was adjusted to 0.3 mg/mL by the addition of the buffered medium. Various xylose concentrations (0.5, 1, 2, and 5 g/L) were used, and the reactions were performed at 37 °C in triplicate. The initial reaction rate of xylose isomerase was calculated as the slope of the linear regression line of the change in the amount of xylulose over time. K_m_ and k_cat_ were calculated using the Lineweaver–Burk equation [73].

### Measurement of synthetic promoter strengths

Cells grown in the LBv2 medium were inoculated into 3 mL of fresh medium in a 15 mL test tube at an OD_600_ of 0.1. When the OD_600_ reached 0.8–1, the culture was transferred to 3 mL of fresh medium at an OD_600_ of 0.1. After 16 h, cells were harvested, and their fluorescence was measured using a Hidex Sense microplate reader (Hidex, Turku, Finland). Specific fluorescence was determined by dividing the measured fluorescence by the OD_600_. Each specific fluorescence was normalized to the value of the VXWP1 strain as a control.

### Sugar and metabolite quantification

Sugars (glucose, xylose, xylulose, and arabinose) and metabolites (lactate, pyruvate, and acetate) were quantified using an UltiMate™ 3000 analytical high-performance liquid chromatography system (Dionex, Sunnyvale, CA, USA) equipped with an Aminex HPX-87H column (Bio-Rad Laboratories). As a mobile phase, we used 5 mM sulfuric acid at a flow rate of 0.6 mL/min and 30 °C. The refractive index signal was monitored using a Shodex RI-101 detector (Shodex, Klokkerfaldet, Denmark).

The overall scheme of this study *Vibrio* sp. dhg was engineered for efficient conversion of lignocellulosic biomass sugars. The VXA0 strain was constructed by heterologous expression of xylose isomerase in *Vibrio* sp. dhg. This heterologous pathway was optimized via the Adaptive Laboratory Evolution (ALE) strategy to construct the VXA38 strain. After enhancing xylose catabolism, CCR was removed for the simultaneous utilization of glucose, xylose, and arabinose to construct the VXA38PG strain. Then, it was further engineered for lactate production to construct the VXA38PGL strain. The resulting strain achieved efficient biochemical production via the rapid and simultaneous utilization of lignocellulose-derived sugars.

## Supplementary Information

**Additional file1 Table S1**. Maximum specific growth rates and sugar consumption rates of *Vibrio* sp. dhg and *E. coli* W grown on glucose, xylose, and arabinose. **Table S2.** List of catabolic genes for sugar utilization in *Vibrio* sp. dhg. **Table S3.** Comparison of carbon and ATP production yields of four known xylose catabolic pathways. **Table S4.** Detailed stoichiometry of the xylose catabolic pathways. **Table S5.** Comparison of xylose utilizing strains from previous studies and VXA38 strains. **Table S6.** Identified mutations in the starting strain, VXA0. **Table S7.** Detailed information on mutations in evolved isolates. **Table S8. **Comparison of lactate production from lignocellulose-derived sugars. **Table S9**. List of strains and plasmids used in this study. **Table S10**. List of primers used in this study**. Table S11.** Detailed information on plasmid construction. **Figure S1.** Ratios of *Vibrio* and *E. coli* species having genes of complete xylose catabolic pathways. **Figure S2.** Known xylose catabolic pathways. **Figure S3.** Growth profiles of the VXA1-1, VXA1-2, VXA15-1, and VXA15-3 strains in xylose minimal medium. **Figure S4.** Sequence alignment of xylulokinase (XylB) from *E. coli* and *Vibrio* sp. dhg. **Figure S5.** Comparison of K_m_ and *k*_cat_ of the wild-type and mutant xylose isomerase. **Figure S6.** Normalized specific fluorescence values of strains expressing the *xylA-sgfp* fused protein with wild-type and mutant promoter and *xylA *coding sequence.

## Data Availability

The data sets used and/or analyzed during the current study are available from the corresponding author on reasonable request.
