# Supplementary material for: A Vibrio-based microbial platform for accelerated lignocellulosic sugar conversion
Source: Biotechnol Biofuels Bioprod. 2022 May 25;15:58. doi: 10.1186/s13068-022-02157-3 (PMC9134653; doi:10.1186/s13068-022-02157-3)
Supplement: Supplementary file 1 — Additional file1 Table S1. Maximum specific growth rates and sugar consumption rates of Vibrio sp. dhg and E. coli W grown on glucose, xylose, and arabinose. Table S2. List of catabolic genes for sugar utilization in Vibrio sp. dhg. Table S3. Comparison of carbon and ATP production yields of four known xylose catabolic pathways. Table S4. Detailed stoichiometry of the xylose catabolic pathways. Table S5. Comparison of xylose utilizing strains from previous studies and VXA38 strains. Table S6. Identified mutations in the starting strain, VXA0. Table S7. Detailed information on mutations in evolved isolates. Table S8. Comparison of lactate production from lignocellulose-derived sugars. Table S9. List of strains and plasmids used in this study. Table S10. List of primers used in this study. Table S11. Detailed information on plasmid construction. Figure S1. Ratios of Vibrio and E. coli species having genes of complete xylose catabolic pathways. Figure S2. Known xylose catabolic pathways. Figure S3. Growth profiles of the VXA1-1, VXA1-2, VXA15-1, and VXA15-3 strains in xylose minimal medium. Figure S4. Sequence alignment of xylulokinase (XylB) from E. coli and Vibrio sp. dhg. Figure S5. Comparison of Km and kcat of the wild-type and mutant xylose isomerase. Figure S6. Normalized specific fluorescence values of strains expressing the xylA-sgfp fused protein with wild-type and mutant promoter and xylA coding sequence. [file 13068_2022_2157_MOESM1_ESM.docx]

**Additional file of**

**A *Vibrio*-based microbial platform for accelerated lignocellulosic sugar conversion**

Sunghwa Woo^a,1^, Hyun Gyu Lim^a,1^, Yong Hee Han^b,1^_,_ Sungwoo Park^c^, Myung Hyun Noh^a^, Dongyeop Baek^a^, Jo Hyun Moon^a^, Sang Woo Seo^b,c,d,e,f,*^, Gyoo Yeol Jung^a,g,*^

^a^Department of Chemical Engineering, Pohang University of Science and Technology, 77 Cheongam-Ro, Nam-Gu, Pohang, Gyeongbuk 37673, Korea

^b^Interdisciplinary Program in Bioengineering, Seoul National University, 1 Gwanak-ro, Gwanak-gu, Seoul 08826, Korea

^c^School of Chemical and Biological Engineering, and ^d^Institute of Chemical Processes, and ^e^Bio-MAX Institute, and ^f^Institute of Engineering Research, Seoul National University, 1 Gwanak-ro, Gwanak-gu, Seoul 08826, Korea

^g^School of Interdisciplinary Bioscience and Bioengineering, Pohang University of Science and Technology, 77 Cheongam-Ro, Nam-Gu, Pohang, Gyeongbuk 37673, Korea

^1^These authors contributed equally.

*To whom correspondence should be addressed.

(Sang Woo Seo)

Mailing address: School of Chemical and Biological Engineering, Seoul National University, 1 Gwanak-Ro, Gwanak-Gu, Seoul 08826, Korea

Tel.: +82-2-880-2274, Fax: +82-2-888-7295, E-mail: swseo@snu.ac.kr

(Gyoo Yeol Jung)

Mailing address: Department of Chemical Engineering, Pohang University of Science and Technology, 77 Cheongam-Ro, Nam-Gu, Pohang, Gyeongbuk 37673, Korea

Tel.: +82-54-279-2391, Fax: +82-54-279-5528, E-mail: gyjung@postech.ac.kr

**Contents**:

Tables S1-S11

Figures S1-S6

References

# Tables

## Table S1. Maximum specific growth rates and sugar consumption rates of *Vibrio* sp. dhg and *E. coli* W grown on glucose, xylose, and arabinose

| **Carbon source** |  | **Maximum specific  growth rate (h^-1^)** | |  | **Maximum specific  sugar uptake rate^a^  (g g_dcw_^-1^ h^-1^)** | |
| --- | --- | --- | --- | --- | --- | --- |
|  |  | ***Vibrio* sp. dhg** | ***E. coli* W** |  | ***Vibrio* sp. dhg** | ***E. coli* W** |
| Glucose |  | 1.31 ± 0.02 | 0.51 ± 0.01 |  | 4.86 ± 0.11 | 1.63 ± 0.05 |
| Arabinose |  | 0.81 ± 0.03 | 0.49 ± 0.01 |  | 3.17 ± 0.12 | 1.69 ± 0.05 |
| Xylose |  | n.d.^a^ | 0.41 ± 0.01 |  | n.d.^a^ | 1.62 ± 0.06 |

^a^n.d., not detected

## Table S2. List of catabolic genes for sugar utilization in *Vibrio* sp. dhg

| **Pathway** | **Gene name** | **Product** | **Location** | | | **Gene size (bp)** |
| --- | --- | --- | --- | --- | --- | --- |
|  |  |  | **Locus tag** | **Start** | **End** |  |
| **Glucose transport** | *ptsG* | PTS system, glucose-specific IIBC component (EC 2.7.1.69) | Chr1.peg.1830 | 2,075,269 | 2,073,839 | 1,431 |
|  | *ptsG* | PTS system, glucose-specific IIBC component (EC 2.7.1.69) | Chr1.peg.1831 | 2,077,829 | 2,076,213 | 1,617 |
|  | *ptsG* | PTS system, glucose-specific IIBC component (EC 2.7.1.69) | Chr2.peg.824 | 917,299 | 915,674 | 1,626 |
|  | *ptsG* | PTS system, glucose-specific IIBC component (EC 2.7.1.69) | Chr2.peg.1396 | 1,570,845 | 1,571,141 | 297 |
| **EMP pathway** | *glk* | Glucokinase (EC 2.7.1.2) | Chr2.peg.1619 | 1,798,907 | 1,799,878 | 972 |
|  | *pgi* | Glucose-6-phosphate isomerase (EC 5.3.1.9) | Chr1.peg.2593 | 2,934,208 | 2,932,556 | 1,653 |
|  | *pfkAB* | 6-phosphofructokinase (EC 2.7.1.11) | Chr1.peg.2700 | 3,049,329 | 3,048,367 | 963 |
|  | glpX | Fructose-1,6-bisphosphatase, GlpX type (EC 3.1.3.11) | Chr1.peg.214 | 236,982 | 235,783 | 1,200 |
|  | *fbp* | Fructose-1,6-bisphosphatase, type I (EC 3.1.3.11) | Chr1.peg.278 | 288,386 | 287,373 | 1,014 |
|  | *fbp* | Fructose-1,6-bisphosphatase, type I (EC 3.1.3.11) | Chr2.peg.592 | 668,816 | 669,826 | 1,011 |
|  | *fbaA* | Fructose-bisphosphate aldolase class II (EC 4.1.2.13) | Chr1.peg.2451 | 2,758,893 | 2,757,817 | 1,077 |
|  | *gapA* | Glyceraldehyde-3-phosphate dehydrogenase (EC 1.2.1.12) | Chr1.peg.1935 | 2,192,349 | 2,193,344 | 996 |
|  | *gapA* | Glyceraldehyde-3-phosphate dehydrogenase (EC 1.2.1.12) | Chr1.peg.2793 | 3,175,045 | 3,174,044 | 1,002 |
|  | *pgk* | Phosphoglycerate kinase (EC 2.7.2.3) | Chr1.peg.2452 | 2,760,208 | 2,759,048 | 1,161 |
|  | *gpmAk* | Phosphoglycerate mutase (EC 5.4.2.1) | Chr1.peg.2678 | 3,027,705 | 3,026,173 | 1,533 |
|  | *eno* | Enolase (EC 4.2.1.11) | Chr1.peg.2417 | 2,722,656 | 2,721,355 | 1,302 |
|  | *pyk* | Pyruvate kinase (EC 2.7.1.40) | Chr1.peg.1824 | 2,067,553 | 2,068,995 | 1,443 |
|  | *pyk* | Pyruvate kinase (EC 2.7.1.40) | Chr1.peg.316 | 331,597 | 330,185 | 1,413 |
|  | *ppsA* | Phosphoenolpyruvate synthase (EC 2.7.9.2) | Chr2.peg.67 | 85,608 | 83,194 | 2,415 |
|  | *ppsA* | Phosphoenolpyruvate synthase (EC 2.7.9.2) | Chr2.peg.839 | 933,008 | 935,395 | 2,388 |
| **ED pathway** | *edd* | Phosphogluconate dehydratase (EC 4.2.1.12) | Chr1.peg.6 | 9,370 | 7,574 | 1,797 |
|  | *eda* | 2-dehydro-3-deoxyphosphogluconate aldolase (EC 4.1.2.14) | Chr2.peg.599 | 675,831 | 675,208 | 624 |
| **Arabinose transport** | *araH* | L-arabinose transport system permease protein | Chr1.peg.1256 | 1,418,332 | 1,419,171 | 840 |
|  | *araF* | L-arabinose-binding periplasmic protein precursor AraF | Chr2.peg.1129 | 1,247,008 | 1,248,006 | 999 |
|  | *araG* | L-arabinose transport ATP-binding protein AraG | Chr2.peg.1130 | 1,248,066 | 1,249,583 | 1,518 |
|  | *araH* | L-arabinose transport system permease protein | Chr2.peg.1131 | 1,249,601 | 1,250,590 | 990 |
| **Arabinose catabolic pathway** | *araA* | L-arabinose isomerase (EC 5.3.1.4) | Chr2.peg.1126 | 1,244,132 | 1,242,630 | 1,503 |
|  | *araB* | Ribulokinase (EC 2.7.1.16) | Chr2.peg.1128 | 1,246,626 | 1,244,923 | 1,704 |
|  | *araB* | Ribulokinase (EC 2.7.1.16) | Chr2.peg.1439 | 1,619,188 | 1,620,789 | 1,602 |
|  | *araD* | L-ribulose-5-phosphate 4-epimerase (EC 5.1.3.4) | Chr2.peg.1127 | 1,244,930 | 1,244,196 | 735 |
| **Xylose transport** | *xylH* | D-xylose transport system permease protein | Chr2.peg.1435 | 1,615,007 | 1,614,018 | 990 |
|  | *xylG* | D-xylose transport ATP-binding protein XylG | Chr2.peg.1436 | 1,616,503 | 1,615,004 | 1,500 |
|  | *xylF* | Xylose ABC transporter, periplasmic xylose-binding protein XylF | Chr2.peg.1437 | 1,617,495 | 1,616,560 | 936 |
| **Xylose catabolic pathway** | *xylB* | Xylulose kinase (EC 2.7.1.17) | Chr1.peg.1685 | 1,867,479 | 1,868,939 | 1,461 |
|  | *xylA* | Xylose isomerase (EC 5.3.1.5) | Not found, queried gene: *xylA* from *E. coli* K-12 MG1655 (NCBI gene ID: 948141) | | | |
|  | *XYL1* | Xylose reductase | Not found, queried gene: *XYL1* from *Pichia stipitis* CBS 6054 (NCBI gene ID: 4839234) | | | |
|  | *XYL2* | Xylitol dehydrogenase | Not found, queried gene: *XYL2* from *Pichia stipitis* CBS 6054 (NCBI gene ID: 4852013) | | | |
|  | *xylB* | Xylose dehydrogenase | Not found, queried gene: *xylB* from *Caulobacter crescentus* NA1000 (NCBI gene ID: 7329904) | | | |
|  | *xylC* | Xylonolactonase | Not found, queried gene: *xylC* from *Caulobacter crescentus* NA1000 (NCBI gene ID: 7329903) | | | |
|  | *xylD* | Xylonate dehydratase | Not found, queried gene: *xylD* from *Caulobacter crescentus* NA1000 (NCBI gene ID: 7329902) | | | |
|  | *xylD* | 2-Keto-3-deoxy xylonate dehydratase | Not found, queried gene: *xylD* from *Caulobacter crescentus* NA1000 (NCBI gene ID: 7329906) | | | |
|  | *xylA* | α-Ketoglutaric semialdehyde dehydrogenase | Not found, queried gene: *xylA* from *Caulobacter crescentus* NA1000 (NCBI gene ID: 7329905) | | | |
|  | *yjhH* | 2-Dehydro-3-deoxy-D-pentonate aldolase | Not found, queried gene: *yjhH* from *E. coli* K-12 MG1655 (NCBI gene ID: 948825) | | | |
|  | *yagE* | 2-Dehydro-3-deoxy-D-pentonate aldolase | Not found, queried gene: *yagE* from *E. coli* K-12 MG1655 (NCBI gene ID: 944925) | | | |
| **Pentose phosphate pathway** | *zwf* | Glucose-6-phosphate 1-dehydrogenase (EC 1.1.1.49) | Chr1.peg.1531 | 1,737,087 | 1,735,585 | 1,503 |
|  | *pgl* | 6-phosphogluconolactonase (EC 3.1.1.31) | Chr1.peg.1530 | 1,735,588 | 1,734,869 | 720 |
|  | *gnd* | 6-phosphogluconate dehydrogenase (EC 1.1.1.44) | Chr1.peg.1529 | 1,734,833 | 1,733,385 | 1,449 |
|  | *rpiA* | Ribose 5-phosphate isomerase A (EC 5.3.1.6) | Chr1.peg.2445 | 2,751,559 | 2,752,215 | 657 |
|  | *rpe* | Ribulose-phosphate 3-epimerase (EC 5.1.3.1) | Chr1.peg.2602 | 2,940,988 | 2,940,317 | 672 |
|  | *tkt* | Transketolase (EC 2.2.1.1) | Chr1.peg.2454 | 2,763,539 | 2,761,545 | 1,995 |
|  | *tkt* | Transketolase (EC 2.2.1.1) | Chr2.peg.690 | 774,939 | 776,930 | 1,991 |
|  | *tal* | Transaldolase (EC 2.2.1.2) | Chr2.peg.689 | 773,897 | 774,856 | 960 |

## Table S3. Comparison of carbon and ATP production yields of four known xylose catabolic pathways

| **Product** |  | **Theoretical maximum yield (mol_product_/mol_xylose_)** | | | | | | |
| --- | --- | --- | --- | --- | --- | --- | --- | --- |
|  |  | **Isomerase** |  | **Oxidoreductase** |  | **Weimberg** |  | **Dahms** |
| Pyruvate^a^ |  | 1.67 |  | 1.67 |  | 1 |  | 1 |
| Ethanol^b^ |  | 1.67 |  | 1.67 |  | 1 |  | 1 |
| 2,3-Butanediol^c^ |  | 0.83 |  | 0.83 |  | 0.5 |  | 0.5 |
| Malate^d^ |  | 0.83 |  | 0.83 |  | 1 |  | 1 |
| ATP^e^ |  | 26.67 |  | 26.67 |  | 25 |  | 24 |

^a^Detailed stoichiometry for pyruvate production is given in Table S4.

^b^Calculated based on the pathway where ethanol is produced from pyruvate via acetaldehyde.

^c^Calculated based on the pathway where 2,3-butanediol is produced from pyruvate via acetolactate and acetoin. Since acetolactate is synthesized from two molecules of pyruvate, it is 50% of the pyruvate yield.

^d^Calculated based on the pathway where acetyl-CoA and oxaloacetate are condensed to citrate and then converted to malate through the TCA cycle. Since one molecule of pyruvate is required for the production of acetyl-CoA and oxaloacetate, respectively, it is 50% of the pyruvate yield.

^e^Calculated by dividing the total moles of produced ATP by the moles of xylose. 1 mole of NADH, FADH2, and GTP was assumed to be equivalent to 2.5, 1.5, and 1 moles of ATP, respectively.

## Table S4. Detailed stoichiometry of the xylose catabolic pathways

| **Summary** | | | |
| --- | --- | --- | --- |
| **Isomerase pathway** | 3 Xylose + 5 ADP + 5 Pi + 5 NAD^+^ | → | 5 Pyruvate + 5 ATP + 5 NADH |
| **Oxido-reductase pathway** | 3 Xylose + 5 ADP + 5 Pi + 8 NAD^+^ + 3 NAD(P)H | → | 5 Pyruvate + 5 ATP + 8 NADH + 3 NAD(P)^+^ |
| **Weimberg pathway** | 3 Xylose + 3 GDP + 3 Pi + 9 NAD^+^ + 3 FAD + 3 NADP^+^ | → | 3 Pyruvate + 3 GTP + 9 NADH + 3 FADH_2_ + 3 NADPH + 6 CO_2_ |
| **Dahms pathway** | 3 Xylose + 12 NAD^+^ + 3 FAD | → | 3 Pyruvate + 12 NADH + 3 FADH_2_ + 6 CO_2_ |
| **Isomerase pathway** | | | |
| **Xylose isomerase** | 3 Xylose | → | 3 Xylulose |
| **Xylulokinase** | 3 Xylulose + 3 ATP | → | 3 Xylulose 5-phosphate + 3 ADP |
| **Ribulose-phosphate 3-epimerase** | 1 Xylulose 5-phosphate | → | 1 Ribulose 5-phosphate |
| **Ribose-5-phosphate isomerase** | 1 Ribulose 5-phosphate | → | 1 Ribose 5-phosphate |
| **Transketolase** | 1 Ribose 5-phosphate + 1 Xylulose 5-phosphate | → | 1 Sedoheptulose 7-phosphate + 1 Glyceraldehyde 3-phosphate |
| **Transaldolase** | 1 Sedoheptulose 7-phosphate + 1 Glyceraldehyde 3-phosphate | → | 1 Erythrose 4-phosphate + 1 Fructose 6-phosphate |
| **Transketolase** | 1 Erythrose 4-phosphate + 1 Xylulose 5-phosphate | → | 1 Fructose 6-phosphate + 1 Glyceraldehyde 3-phosphate |
| **Phosphofructokinase** | 2 Fructose 6-phosphate + 2 ATP | → | 2 Fructose 1,6-bisphosphate + 2 ADP |
| **Fructose-bisphosphate aldolase** | 2 Fructose 1,6-bisphosphate | → | 2 Dihydroxyacetone phosphate + 2 Glyceraldehyde 3-phosphate |
| **Triose-phosphate isomerase** | 2 Dihydroxyacetone phosphate | → | 2 Glyceraldehyde 3-phosphate |
| **Glyceraldehyde-3-phosphate dehydrogenase** | 5 Glyceraldehyde 3-phosphate + 5 P_i_ + 5 NAD^+^ | → | 5 3-Phosphoglyceroyl phosphate + 5 NADH |
| **3-Phosphoglycerate kinase** | 5 3-Phosphoglyceroyl phosphate + 5 ADP | → | 5 3-Phosphoglycerate + 5 ATP |
| **Phosphoglycerate mutase** | 5 3-Phosphoglycerate | → | 5 2-Phosphoglycerate |
| **Enolase** | 5 2-Phosphoglycerate | → | 5 Phosphoenolpyruvate |
| **Pyruvate kinase** | 5 Phosphoenolpyruvate + 5 ADP | → | 5 Pyruvate + 5 ATP |
| **Net** | **3 Xylose + 5 ADP + 5 P_i_ + 5 NAD^+^** | → | **5 Pyruvate + 5 ATP + 5 NADH** |
|  |  |  |  |
| **Oxido-reductase pathway** | | | |
| **Xylose reductase** | 3 Xylose + 3 NAD(P)H | → | 3 Xylitol + 3 NAD(P)+ |
| **Xylitol dehydrogenase** | 3 Xylitol + 3 NAD^+^ | → | 3 Xylulose + 3 NADH |
| **Xylulokinase** | 3 Xylulose + 3 ATP | → | 3 Xylulose 5-phosphate + 3 ADP |
| **Ribulose-phosphate 3-epimerase** | 1 Xylulose 5-phosphate | → | 1 Ribulose 5-phosphate |
| **Ribose-5-phosphate isomerase** | 1 Ribulose 5-phosphate | → | 1 Ribose 5-phosphate |
| **Transketolase** | 1 Ribose 5-phosphate + 1 Xylulose 5-phosphate | → | 1 Sedoheptulose 7-phosphate + 1 Glyceraldehyde 3-phosphate |
| **Transaldolase** | 1 Sedoheptulose 7-phosphate + 1 Glyceraldehyde 3-phosphate | → | 1 Erythrose 4-phosphate + 1 Fructose 6-phosphate |
| **Transketolase** | 1 Erythrose 4-phosphate + 1 Xylulose 5-phosphate | → | 1 Fructose 6-phosphate + 1 Glyceraldehyde 3-phosphate |
| **Phosphofructokinase** | 2 Fructose 6-phosphate + 2 ATP | → | 2 Fructose 1,6-bisphosphate + 2 ADP |
| **Fructose-bisphosphate aldolase** | 2 Fructose 1,6-bisphosphate | → | 2 Dihydroxyacetone phosphate + 2 Glyceraldehyde 3-phosphate |
| **Triose-phosphate isomerase** | 2 Dihydroxyacetone phosphate | → | 2 Glyceraldehyde 3-phosphate |
| **Glyceraldehyde-3-phosphate dehydrogenase** | 5 Glyceraldehyde 3-phosphate + 5 Pi + 5 NAD^+^ | → | 5 3-Phosphoglyceroyl phosphate + 5 NADH |
| **3-Phosphoglycerate kinase** | 5 3-Phosphoglyceroyl phosphate + 5 ADP | → | 5 3-Phosphoglycerate + 5 ATP |
| **Phosphoglycerate mutase** | 5 3-Phosphoglycerate | → | 5 2-Phosphoglycerate |
| **Enolase** | 5 2-Phosphoglycerate | → | 5 Phosphoenolpyruvate |
| **Pyruvate kinase** | 5 Phosphoenolpyruvate + 5 ADP | → | 5 Pyruvate + 5 ATP |
| **Net** | 3 Xylose + 5 ADP + 5 Pi + 8 NAD^+^ + 3 NAD(P)H | → | 5 Pyruvate + 5 ATP + 8 NADH + 3 NAD(P)^+^ |
|  |  |  |  |
| **Weimberg pathway** | | | |
| **Xylose dehydrogenase** | Xylose + NAD^+^ | → | Xylono-1,4-lactone + NADH |
| **Xylonolactonase** | Xylono-1,4-lactone | → | Xylonate |
| **Xylonate dehydratase** | Xylonate | → | 2-Dehydro-3-deoxy-xylonate |
| **2-Dehydro-3-deoxy-xylonate dehydratase** | 2-Dehydro-3-deoxy-xylonate | → | α-Ketoglutarate semialdehyde |
| **α-Ketoglutaric semialdehyde dehydrogenase** | α-Ketoglutarate semialdehyde + NADP^+^ | → | α-Ketoglutarate + NADPH |
| **α-Ketoglutarate dehydrogenase** | α-Ketoglutarate + NAD^+^ + CoA-SH | → | Succinyl-CoA + CO_2_ + NADH |
| **Succinyl-CoA synthase** | Succinyl-CoA + GDP + Pi | → | Succinate + GTP + CoA-SH |
| **Succinate dehydrogenase** | Succinate + FAD | → | Fumarate + FADH_2_ |
| **Fumarase** | Fumarate | → | Malate |
| **Malate dehydrogenase** | Malate + NAD^+^ | → | Oxaloacetate + NADH |
| **Phosphoenolpyruvate carboxykinase** | Oxaloacetate + ATP | → | Phosphoenolpyruvate + ADP + CO_2_ |
| **Pyruvate kinase** | Phosphoenolpyruvate + ADP | → | Pyruvate + ATP |
| **Net** | 1 Xylose + 1 GDP + 1 Pi + 3 NAD^+^ + 1 FAD + 1 NADP^+^ | → | 1 Pyruvate + 1 GTP + 3 NADH + 1 FADH_2_ + 1 NADPH + 2 CO_2_ |
| **Dahms pathway** | | | |
| **Xylose dehydrogenase** | Xylose + NAD^+^ | → | Xylono-1,4-lactone + NADH |
| **Xylonolactonase** | Xylono-1,4-lactone | → | Xylonate |
| **Xylonate dehydratase** | Xylonate | → | 2-Dehydro-3-deoxy-xylonate |
| **2-Dehydro-3-deoxy-pentonate aldolase** | 2-Dehydro-3-deoxy-xylonate | → | Pyruvate + Glycoaldehyde |
| **Pyruvate dehydrogenase** | Pyruvate + CoA-SH + NAD^+^ | → | Acetyl-CoA + NADH + CO_2_ |
| **Glycolaldehyde dehydrogenase** | Glycolaldehyde + NAD^+^ | → | Glycolate + NADH |
| **Glycolate dehydrogenase** | Glycolate + FAD | → | Glyoxylate + FADH_2_ |
| **Malyl-CoA lyase** | Acetyl-CoA + Glyoxylate | → | Malyl-CoA |
| **Malyl-CoA thioesterase** | Malyl-CoA | → | Malate + CoA-SH |
| **Malate dehydrogenase** | Malate + NAD^+^ | → | Oxaloacetate + NADH |
| **Phosphoenolpyruvate carboxykinase** | Oxaloacetate + ATP | → | Phosphoenolpyruvate + ADP + CO_2_ |
| **Pyruvate kinase** | Phosphoenolpyruvate + ADP | → | Pyruvate + ATP |
| **Net** | 1 Xylose + 4 NAD^+^ + 1 FAD | → | 1 Pyruvate + 4 NADH + 1 FADH_2_ + 2 CO_2_ |

## Table S5. Comparison of xylose utilizing strains from previous studies and VXA38 strains

| **Host strain** | **Type**  **(wildtype or engineered)** | **Specific growth rate on xylose  (h^-1^)** | **Specific xylose consumption rate (g g_dcw_^-1^ h^-1^)** | **Culture condition** | **Reference** |
| --- | --- | --- | --- | --- | --- |
| *Pichia stipitis* | Wildtype | 0.12 | 0.10 | Rich medium, aerobic | [1] |
| *Clostridium tyrobutyricum* | Wildtype | 0.12 | n.d. | Defined medium, anaerobic | [2] |
| *Corynebacterium glutamicum* | Engineered | 0.07 | n.d. | Defined medium, aerobic | [3] |
| *Saccharomyces cerevisiae* | Engineered | 0.20 | 0.70 | Rich medium, anaerobic | [4] |
| *Pseudomonas putida* KT2440 | Engineered | 0.21 | n.d. | Defined medium, aerobic | [5] |
| *E. coli* | Engineered | 0.34 | 0.94 | Defined medium, aerobic | [6] |
| *Vibrio* sp. dhg | Engineered | 0.67 | 2.15 | Defined medium, aerobic | This study |

## Table S6. Identified mutations in the starting strain, VXA0

| **Position** | **Mutation** | **Gene product** |
| --- | --- | --- |
| **Chromosome 1** | | |
| 190,931 | +^a^ | Glycosyl transferase domain protein |
| 721,562 | A147T (**G**CG→**A**CG) | Thiamine biosynthesis protein *thiI* |
| 975,004 | +G | tRNA-Tyr-GTA |
| 1,015,314 | C5S (T**G**T→T**C**T) | Thiamine kinase (EC 2.7.1.89) Adenosylcobinamide kinase (EC 2.7.1.156) |
| 1,746,440 | G→A | Intergenic region |
| 1,821,426 | (GATT)_2→1_ | Intergenic region |
| 2,768,312 | N434N (AA**T**→AA**C**) | Xylose isomerase (EC 5.3.1.5) |
| **Chromosome 2** | | |
| 22,916 | P87P (CC**G**→CC**C**) | Methylcrotonyl-CoA carboxylase carboxyl transferase subunit (EC 6.4.1.4) |
| 646,893 | (G)_5→6_ | Predicted hydrolase |
| 1,456,700 | Δ(CTGTTGCTG) | TPR domain protein in aerotolerance operon |
| **Plasmid** | | |
| 51,986 | (G)_6→7_ | Aconitate hydratase (EC 4.2.1.3) |
| 54,467 | (G)_10→11_ | Intergenic region |
| 129,285 | C→T | Intergenic region |
| 129,561 | E4E (GA**A**→GA**G**) | Mobile element protein |

^a^A large insertion. The inserted sequence is as follow:
cagggtaagattacgaagccccttgccccgcaaggactttggaaagataagcgttacttcgattcgctttcttctgtttccgcttaatgcctgctttaaagcttgtctctgccgttaacaggtttaacgacgcatgacggataaccgcgaagttctctcccgcttgctctcgcctgattcgacaattatcttcattaaagccaacatccaaacgccaatgcatttgattctcgatgctccagtgcgagcgcgtcccttctagcaactcttttgcgctcaatttagctgagctaatataatgctttatctgcattgtctccgctggcttattaccctcttgtcgaattgaaacaactacaccaagcgttgttagttgagaccactcaaaagccaaatcaccgagtaagtcaacgttatgctccacaaggcaaaggcgtgtttctgtacgtccatgccccttttcttgaacgacaatcttgtcgccctcaaatgagttcaacatggtcgcgttaaatatctcgttcatcgcatggttaagctttttttgattgtctttaaccgctaagagataatcggcttctttcctaattattttatttgcaatcgatttttggcaacccatcgcatcgatagtcactaagcaacccttgatagataggagctccagcagctcggggatcgctgtgatctcattacttttctcgcttgtttttacctgacccaacaccatcttgttagcagcactaaaagcgctaaccatatgaatcgctccactacgcttgtctttgttgtaggtaccgcggagcgtcttaccatcgattgcgacaacttcgccttcagtcgcctcatgacagtctttcatccactgggcaaagcagcgctgcaactgcttggctgagataagattaataactcttgctatggtgtcatgaacagggactccattttcaaagtcaccatattgcctgagccaatcaaggttatcttgaccaaagtcttcgatatcttcccacccttctgcgccagcaatgacagcagcaaccataaggaaaatgatatctgtcagtttgtgttcaaccttccatgattggcgagggtcacttattaccgagatatgttctaacaagcttgctccagtcatctgatttactcccaaaagagagtatatgatcacgactaaacatgatcgtcaaatcgatccttaattttgttactatttcgatgcattcattatactttgggtgtaattcttacttgtgctgaccccttattccataagggtttttcatgatcttgccctgaggaagctcg

## Table S7. Detailed information of mutations in evolved isolates

| **Location** | **Gene** | **DNA modification** | **Protein modification** |
| --- | --- | --- | --- |
| Chr2; 1,846,276 | *yrkL* | [chromosome 2 ref.]: g.1846276_1847699delinsT | T55X |
|  | *deoR* |  | V55HfsX2 |
| Chr2; 1,409,023 | *scrC* | [scrC ref.]: c.95C>T | T32I |
| Chr1; 2,768,325 | *xylA* | [xylA ref.]: c.8C>A | A3D |
| Chr1; 2,768,285 |  | [xylA ref.]: c.-35C>A | - |
| Chr1; 2,875,221 | *cafA* | [cafA ref.]: c.203T>A | L68X |

Each mutation is represented by a HGVS nomenclature [7].

Each letter prefix indicates the type of reference sequence used; g., a linear genomic reference sequence; c., a coding DNA reference sequence. X indicates the generation of a stop codon.

## Table S8. Comparison of lactate production from lignocellulose-derived sugars

| **Strain** | **Substrate** | **Strategy** | **Titer  (g/L)** | **Y_p/s_  (g/g)** | **q_s_ (g/L/h)** | **Reference** |
| --- | --- | --- | --- | --- | --- | --- |
|  |  |  |  |  |  |  |
| *Lactobacillus plantarum* Δ*ldhL*::*PxylAB-xpk1*::*tkt*Δ*xpk2*::*PxylAB* | Glucose:xylose:arabinose =10:5:1 | Δ*ldhL*::*PxylAB-xpk1*::*tkt*Δ*xpk2*::*PxylAB* | 61 | 0.79 | 2.35 | [8] |
| *Lactococcus lactis IO-1* | Xylose | An isolate | 33 | 0.67 | 0.67 | [9] |
| *E. coli* B WL204 | Xylose | Δ*frdBC* Δ*ldhA* Δ*ackA* Δ*pflB* Δ*pdhR* Δ*mgsA* Δ*adhE* Δ*ldhA*::*ldhL* (from *Pediococcus acidilactici*) Evolved in anaerobic condition | 66 | 0.90 | 1.09 | [10] |
| *E. coli* B FBR19 | Glucose:xylose =1:1 | Δ*ptsG* Δ*frdABCD* *ldhL*↑ (*Streptococcus bovis*) | 64 | 0.77 | 0.43 | [11] |
| *E. coli* B JH15 | Glucose:xylose:arabinose=6:3:1 | Δ*frdBC* Δ*ldhA* Δ*ackA* Δ*pflB* Δ*pdhR* Δ*mgsA* Δ*adhE* Δ*ptsG* *ldhA* ↑ (*E. coli*) Evolved in anaerobic condition | 81 | 0.81 | 0.81 | [12] |
| *Vibrio* sp. dhgVXA38PGL | Glucose:xylose:arabinose=6:3:1 | Δ*ptsG* Δ*frdABCD* Δ*pflB ldhA* ↑ (*Vibrio* sp. dhg) | 83 | 0.80 | 1.15 | This study |

## Table S9. List of strains and plasmids used in this study

| **Name** | **Relevant characteristics** | **Source** |
| --- | --- | --- |
| Strains |  |  |
| Mach-T1^R^ | *E. coli* F^-^ φ80(*lacZ*)ΔM15 Δ*lac*X74 *hsd*R(rK^-^mK^+^) Δ*rec*A1398 *end*A1 *ton*A | Invitrogen |
| *Vibrio* sp. dhg | A novel alginate-metabolizing microorganism | [13] |
| *Escherichia coli* W | Source of xylose isomerase gene *(xylA*) | ATCC 9637 |
| VXA0 | *Vibrio* sp. dhg Δ*dns*::*xylA*; the parental strain of evolved strain | This study |
| VXA1-1 | 1^st^ isolated strain from 1^st^ passage | This study |
| VXA1-2 | 2^nd^ isolated strain from 1^st^ passage | This study |
| VXA1-3 | 3^rd^ isolated strain from 1^st^ passage | This study |
| VXA3-1 | 1^st^ isolated strain from 3^rd^ passage | This study |
| VXA3-2 | 2^nd^ isolated strain from 3^rd^ passage | This study |
| VXA3-3 | 3^rd^ isolated strain from 3^rd^ passage | This study |
| VXA15-1 | 1^st^ isolated strain from 15^th^ passage | This study |
| VXA15-2 | 2^nd^ isolated strain from 15^th^ passage | This study |
| VXA15-3 | 3^rd^ isolated strain from 15^th^ passage | This study |
| VXA38-1 | 1^st^ isolated strain from 38^th^ passage | This study |
| VXA38-2 | 2^nd^ isolated strain from 38^th^ passage | This study |
| VXA38-3 | 3^rd^ isolated strain from 38^th^ passage | This study |
| VXA38C | VXA38-1/pACYC_Duet | This study |
| VXA38Y | VXA38-1/pACYC_yrkL | This study |
| VXA38D | VXA38-1/pACYC_deoR | This study |
| VXW | *Vibrio* sp. dhg /pACYC_xylAWT_Histag | This study |
| VXM | *Vibrio* sp. dhg /pACYC_xylAMUT_Histag | This study |
| VXWP1 | *Vibrio* sp. dhg /pACYC_PWT_xylAWT_sgfp | This study |
| VXMP1 | *Vibrio* sp. dhg /pACYC_PWT_xylAMUT_sgfp | This study |
| VXWP2 | *Vibrio* sp. dhg /pACYC_PMUT_xylAWT_sgfp | This study |
| VXMP2 | *Vibrio* sp. dhg /pACYC_PMUT_xylAMUT_sgfp | This study |
| VXA38P | VX38-1Δ*ptsG* | This study |
| VXA38PG | VXA38P/pACYC_galP | This study |
| VXA38PGL | VXA38PΔ*frdABCD*Δ*pflB*/pACYC_galP_ldhA | This study |
|  |  |  |
| Plasmids |  |  |
| pACYC_Duet | p15A, LacI, Cm^R^ |  |
| pCDF_Duet | CloDF13, LacI, Sm^R^ | Novagen |
| FRT72_cat | T-vector, containing *cat* gene flanked by FRT variant sequences^2^ | [13] |
|  | <https://benchling.com/s/seq-4haQ2Gxa3aKmeDzZ4xTE> |  |
| pACYA_SXT | p15A, Amp^R^, SXT recombinase expression vector | [13] |
|  | <https://benchling.com/s/seq-FhfIXerQDFHIt9gIt9As> |  |
| pRSF_FLP | RSF1030, P_VP13__synUTR_*flp*^SC^ | [13] |
|  | <https://benchling.com/s/seq-jY7lWWd7Gsty3xBlHlTc> |  |
| pCDF_xylA_ins | CloDF13, Sm^R^, homology(*dns*)-Cm^R^-P_J23100__synUTR_*xylA*-homology(*dns*) | This study |
|  | <https://benchling.com/s/seq-DA74jUxzmRdXB5dKUIu4> |  |
| pCDF_ptsG_del | CloDF13, Sm^R^, homology(*ptsG*)-Cm^R^-homology(*ptsG*) | This study |
|  | <https://benchling.com/s/seq-VwAS0kZdqJKVELRRLpS5> |  |
| pCDF_frdABCD_del | CloDF13, Sm^R^, homology(*frdABCD*)-Cm^R^-homology(*frdABCD*) | [13] |
|  | <https://benchling.com/s/seq-F0lDcXn6Qms6sPDedCsk> |  |
| pCDF_pflB_del | CloDF13, Sm^R^, homology(*pflB*)-Cm^R^-homology(*pflB*) | [13] |
|  | <https://benchling.com/s/seq-6X4R44kfy2lhdUv33mGc> |  |
| pACYC_yrkL | p15A, Cm^R^, *yrkL* | This study |
|  | <https://benchling.com/s/seq-qR5MJFhWQU838pYZYH6I> |  |
| pACYC_deoR | p15A, Cm^R^, *deoR* | This study |
|  | <https://benchling.com/s/seq-Zyk7VYPkUQPdoWluvVcI> |  |
| pACYC_xylAWT_Histag | p15A, Cm^R^, P_J23100__synUTR_*xylA*^WT^_His tag_BBa_B1001 terminator | This study |
|  | <https://benchling.com/s/seq-DclQppeaUXJnhlKV5Oh9> |  |
| pACYC_xylAMUT_Histag | p15A, Cm^R^, P_J23100__synUTR_*xylA*^MUT^(A3D)_His tag_BBa_B1001 terminator | This study |
|  | <https://benchling.com/s/seq-DclQppeaUXJnhlKV5Oh9> |  |
| pACYC_PWT_xylAWT_sgfp | p15A, Cm^R^, P_J23100__synUTR_ *xylA*^WT^ _*sgfp*_BBa_B1001 terminator | This study |
|  | https://benchling.com/s/seq-Y9iwkqW44YN13T6dxfbh |  |
| pACYC_PWT_xylAMUT_sgfp | p15A, Cm^R^, P_J23100__synUTR_ *xylA*^MUT^(A3D)_*sgfp*_BBa_B1001 terminator | This study |
|  | https://benchling.com/s/seq-Y9iwkqW44YN13T6dxfbh |  |
| pACYC_PMUT_xylAWT_sgfp | p15A, Cm^R^, P_mutant__synUTR_ *xylA*^WT^ _*sgfp*_BBa_B1001 terminator |  |
|  | https://benchling.com/s/seq-Y9iwkqW44YN13T6dxfbh |  |
| pACYC_PMUT_xylAMUT_sgfp | p15A, Cm^R^, P_mutant__synUTR_ *xylA*^MUT^(A3D)_*sgfp*_BBa_B1001 terminator |  |
|  | https://benchling.com/s/seq-Y9iwkqW44YN13T6dxfbh |  |
| pACYC_galP | p15A, Cm^R^, P_J23100__synUTR_*galP*_BBa_B1001 terminator | This study |
|  | <https://benchling.com/s/seq-nBFLelMWJifAR7LuDPnw> |  |
| pACYC_galP_ldhA | p15A, Cm^R^, P_J23100__synUTR_*galP*_BBa_B1001 terminator, P_J23100__synUTR_*ldhA*_BBa_B1001 terminator | This study |
|  | https://benchling.com/s/seq-V0IdnM8ZFiMhqhqhUYXH |  |
|  |  |  |

## Table S10. List of primers used in this study

| **Name** | **Sequence (5’-3’)** |
| --- | --- |
| pCDF_xylA_ins_F1 | ctacgaaatccactacatggccaccgctgagcaataacta |
| pCDF_xylA_ins_R1 | caaaccgcaagcagaaccgcaaattgcactgaaatctagagc |
| pCDF_xylA_ins_F2 | tctagatttcagtgcaatttgcggttctgcttgcggtttg |
| pCDF_xylA_ins_R2 | tcgcatcgcgccggtcatgctatttcctattgagacttgattgaactgag |
| pCDF_xylA_ins_F3 | tcaagtctcaataggaaatagcatgaccggcgcgatgcga |
| pCDF_xylA_ins_R3 | aggactgagctagccgtcaagctcagcggatctcatgcgc |
| pCDF_xylA_ins_F4 | gcgcatgagatccgctgagcttgacggctagctcagtcct |
| pCDF_xylA_ins_R4 | atcatcgcgattggtgaggaaaaaaaaaaccccgccgaag |
| pCDF_xylA_ins_F5 | cttcggcggggttttttttttcctcaccaatcgcgatgat |
| pCDF_xylA_ins_R5 | tagttattgctcagcggtggccatgtagtggatttcgtagc |
| pCDF_ptsG_del_F1 | gtcacccctacagagagtaaccaccgctgagcaataacta |
| pCDF_ptsG_del_R1 | accattggccgattgagaagaaattgcactgaaatctagagc |
| pCDF_ptsG_del_F2 | tctagatttcagtgcaatttcttctcaatcggccaatggt |
| pCDF_ptsG_del_R2 | tcgcatcgcgccggtcatgcggtggtgttcaggctatctt |
| pCDF_ptsG_del_F3 | aagatagcctgaacaccaccgcatgaccggcgcgatgcga |
| pCDF_ptsG_del_R3 | tctttagtaaactttcgcaagctcagcggatctcatgcgcg |
| pCDF_ptsG_del_F4 | gcgcatgagatccgctgagcttgcgaaagtttactaaagaaac |
| pCDF_ptsG_del_R4 | tagttattgctcagcggtggttactctctgtaggggtgac |
| xylA_ch_F | gaataccgatgtcgctgcac |
| xylA_ch_R | ttcgagggatacgcatgaga |
| xylA_1K_F | ggttctgcttgcggtttgta |
| xylA_1K_R | ttgcgcttcaagcatcatgg |
| ptsG_ch_F | tcacgtatgaggttagagcacg |
| ptsG_ch_R | caggagcaaaactaatgaaagg |
| ptsG_1K_F | tgctctatttaccgacggctac |
| ptsG_1K_R | ggtgactctggactcgtttc |
| frdABCD_ch_F | cctatgcgctcacacacggtc |
| frdABCD_ch_R | cgcactgataaggccagcgaac |
| frdABCD_1K_F | ctggattgtggtaacggccattctc |
| frdABCD_1K_R | gaaaacggtaccggtcgtagacaaaac |
| pflB_ch_F | ggtgactctggactcgtttc |
| pflB_ch_R | cctatgcgctcacacacggtc |
| pflB_1K_F | cgcactgataaggccagcgaac |
| pflB_1K_R | ctggattgtggtaacggccattctc |
| yrkL_F | tactacggtacccgaccccagatgcaccaatgac |
| yrkL_R | atcatcgcggccgccgcaataagcgaattttcagtttaaacagtagc |
| deoR_F | tactacggtacctaattgatcaaattaccaaataaagagcgatagatcacaaatgagc |
| deoR_R | atcatcgcggccgccacactaatttggctaaacgtttctaccatattaattaaacacattaac |
| galP_F1 | atgcctgacgctaaaaaacag |
| galP_R1 | ttaatcgtgagcgccgatttc |
| galP_F2 | ttgacggctagctcagtcctaggtacagtgctagcaaacgacggtcgaaaggagggggagatgcctgacgctaaaaaacag |
| galP_R2 | aaaaaaaaaccccgccgaagcggggttttttttttatgatttaatcgtgagcgccgatttc |
| xylA_His_F1 | atgcaagcctattttgaccagctcgatc |
| xylA_His_R1 | tttgtcgaacagataatgatttaccagattttccagttg |
| xylA_His_F2 | ttgacggctagctcagtcctaggtacagtgctagcaagtgaaacctagaaggagatctgtatgcaagcctattttgaccagctcgatc |
| xylA_His_R2 | aaaaaaaaaccccgccgaagcggggttttttttttatgatttagtggtgatgatggtgatgtttgtcgaacagataatgatttaccagattttccagttg |
| pACYC_ins_F | caccggaaggagctgactgggttgaaggcttgacggctagctcagtcctaggtac |
| pACYC_ins_R | gcgtcgagatcctgatagttcgcgttaaaaaaaaaccccgccgaagc |
| pACYC_vec_F | cgcttcggcggggtttttttttaacgcgaactatcaggatctcgacg |
| pACYC_vec_R | gtacctaggactgagctagccgtcaagccttcaacccagtcagctccttc |
| xylAMUT_sdm_F | ctattttgaccagctcgatcgcgtt |
| xylAMUT_sdm_R | tcttgcatacagatctccttctaggtttcac |
| pACYC_sgfp_ins_F | caactggaaaatctggtaaatcattatctgttcgacaaaggcggtgggtcgggcggtgggtccggcggtgggagtggcggtgggagcatggcaagtaaaggagaagaattattcacaggag |
| pACYC_sgfp_ins_R | gtcgcataagggagagcgtcgagatcctgatagttcgcgttaaaaaaaaaccccgccgaagcggggttttttttttatgatttacttgtacagctcgtccatgccgag |
| pACYC_sgfp_vec_F | tctcgacgctctcccttatgcgac |
| pACYC_sgfp_vec_R | tttgtcgaacagataatgatttaccagattttccagttg |
| pMUT_sdm_F | gatctccttctaggtttcacttgctagcact |
| pMUT_sdm_R | ttacctaggactgagctagccgtcaag |
| ldhA_F1 | atgattaacattgctttttttagcgccaaatcctatg |
| ldhA_R1 | ttagttgatcagttcattgcctgaaacttgc |
| ldhA_F2 | ttgacagctagctcagtcctaggtataatgctagcaaaaaacacaaaaggagcatcaagaatgattaacattgctttttttagcgccaaatcctatg |
| ldhA_R2 | aaaaaaaaaccccgccgaagcggggttttttttttatgatttagttgatcagttcattgcctgaaacttgc |
| pACYC_ldhA_ins_F | ccttatgcgactcctgcattaggaaatttgacagctagctcagtcctaggtataatgc |
| pACYC_ldhA_ins_R | tgaccgtgtgcttctcaaatgcctgaggtttcagaaaaaaaaaccccgccgaagcg |
| pACYC_ldhA_vec_F | ccgcttcggcggggtttttttttctgaaacctcaggcatttgagaagcac |
| pACYC_ldhA_vec_R | gctagcattatacctaggactgagctagctgtcaaatttcctaatgcaggagtcgcataagg |

underline indicates overhang sequence

## Table S11. Detailed information on plasmid construction

| **Plasmid** | **Cloning method** | **Fragment** | **Contents** | **Method** | **Primers** | **Template** |
| --- | --- | --- | --- | --- | --- | --- |
| pCDF_xylA_ins | Gibson assembly | 1 | cloDF13, SmR | PCR | pCDF_xylA_ins_F1/R1 | pCDF_Duet plasmid |
|  |  | 2 | 1st homology of *dns* | PCR | pCDF_xylA_ins_F2/R2 | *Vibrio* sp. dhg gDNA |
|  |  | 3 | FRT_cat_FRT | PCR | pCDF_xylA_ins_F3/R3 | pCDF_frdABCD_del plasmid |
|  |  | 4 | PJ23100_synUTR_*xylA*_BBa_B1001 terminator | PCR | xylA_F1/R1 | *E. coli* W gDNA |
|  |  |  |  | PCR | xylA_F2/R2 |  |
|  |  |  |  | PCR | pCDF_xylA_ins_F4/R4 |  |
|  |  | 5 | 2nd homology of *dns* | PCR | pCDF_xylA_ins_F5/R5 | *Vibrio* sp. dhg gDNA |
| pCDF_ptsG_del | Gibson assembly | 1 | cloDF13, SmR | PCR | pCDF_ptsG_del_F1/R1 | pCDF_Duet plasmid |
|  |  | 2 | 1st homology of *ptsG* | PCR | pCDF_ptsG_del_F2/R2 | *Vibrio* sp. dhg gDNA |
|  |  | 3 | FRT_cat_FRT | PCR | pCDF_ptsG_del_F3/R3 | pCDF_frdABCD_del plasmid |
|  |  | 4 | 2nd homology of *ptsG* | PCR | pCDF_ptsG_del_F4/R4 | *Vibrio* sp. dhg gDNA |
| pACYC_yrkL | Restriction /ligation | 1 | p15A, CmR | Restriction enzyme cut of pACYC_duet plasmid | | |
|  |  | 2 | *yrkL* | PCR | yrkL_F | *Vibrio* sp. dhg gDNA |
| pACYC_deoR | Restriction /ligation | 1 | p15A, CmR, | Restriction enzyme cut of pACYC_duet plasmid | | |
|  |  | 2 | *deoR* | PCR | deoR_F | *Vibrio* sp. dhg gDNA |
| pACYC_xylAWT_Histag | Gibson assembly | 1 | p15A, CmR | PCR | pACYC_vec_F/R | pACYC_duet plasmid |
|  |  | 2 | PJ23100_synUTR_*xylA*WT_Histag_BBa_B1001 terminator | PCR | xylA_His_F1/R1 | *E. coli* W gDNA |
|  |  |  |  | PCR | xylA_His_F2/R2 |  |
|  |  |  |  | PCR | pACYC_ins_F/R |  |
| pACYC_xylAMUT_Histag | Site directed mutagenesis | 1 | p15A, CmR, PJ23100_synUTR_xylAMUT(A3D)_His tag_BBa_B1001 terminator | PCR | xylAMUT_sdm_F/R | pACYC_xylAWT_Histag plasmid |
| pACYC_PWT_xylAWT_sgfp | Gibson assembly | 1 | p15A, CmR, PJ23100_synUTR_xylAWT_BBa_B1001 terminator | PCR | pACYC_sgfp_vec_F/R | pACYC_xylAWT_Histag plasmid |
|  |  | 2 | GGGS linker_sgfp | PCR | pACYC_sgfp_ins_F/R | Synthesized sgfp coding sequence using the gBlock^R^ (Integrated DNA Technologies, Skokie, USA) |
| pACYC_PWT_xylAMUT_sgfp | Gibson assembly | 1 | p15A, CmR, PJ23100_synUTR_xylAMUT(A3D)_BBa_B1001 terminator | PCR | pACYC_sgfp_vec_F/R | pACYC_xylAMUT_Histag plasmid |
|  |  | 2 | GGGS linker_sgfp | PCR | pACYC_sgfp_ins_F/R | Synthesized sgfp coding sequence using the gBlock^R^ (Integrated DNA Technologies, Skokie, USA) |
| pACYC_PMUT_xylAWT_sgfp | Site directed mutagenesis | 1 | p15A, CmR, Pmutant_synUTR_xylAWT_sgfp_BBa_B1001 terminator | PCR | pMUT_sdm_F/R | pACYC_PWT_xylAWT_sgfp plasmid |
| pACYC_PMUT_xylAMUT_sgfp | Site directed mutagenesis | 1 | p15A, CmR, Pmutant_synUTR_xylAMUT(A3D)_sgfp_BBa_B1001 terminator | PCR | pMUT_sdm_F/R | pACYC_PWT_xylAMUT_sgfp plasmid |
| pACYC_galP | Gibson assembly | 1 | p15A, CmR, | PCR | pACYC_vec_F/R | pACYC_duet plasmid |
|  |  | 2 | PJ23100_synUTR_galP_BBa_B1001 terminator | PCR | galP_F1/R1 | *E. coli* W gDNA |
|  |  |  |  | PCR | galP_F2/R2 |  |
|  |  |  |  | PCR | pACYC_ins_F/R |  |
| pACYC_galP_ldhA | Gibson assembly | 1 | p15A, CmR, PJ23100_synUTR_galP_BBa_B1001 terminator | PCR | pACYC_ldhA_vec_F/R | pACYC_galP plasmid |
|  |  | 2 | PJ23100_synUTR_ldhA_BBa_B1001 terminator | PCR | ldhA_F1/R1 | *Vibrio* sp dhg gDNA |
|  |  |  |  | PCR | ldhA_F2/R2 |  |
|  |  |  |  | PCR | pACYC_ldhA_ins_F/R |  |

# Figures

## Figure S1. Ratios of *Vibrio* and *E. coli* species having genes of complete xylose catabolic pathways


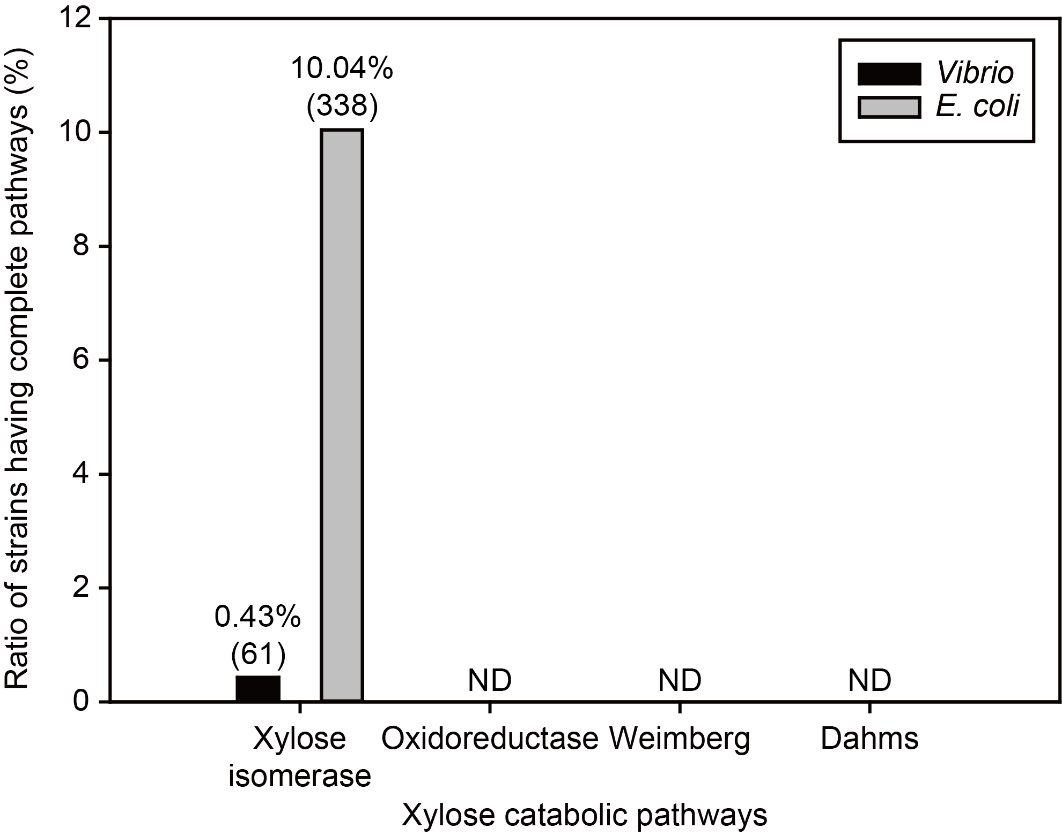


The existence of the four xylose catabolic pathways was investigated in genomes of *Vibrio* (*n* = 14,153; tax id 662) and *E. coli* (*n* = 3,366; tax id 562) deposited at National Center for Biotechnology Information (NCBI, <https://www.ncbi.nlm.nih.gov/>) by using Position-Specific Iterated BLAST (PSI-BLAST). The query protein sequences were from *E. coli* W (accession number CP002185), *Pichia* *stipitis* (accession number NC_009046), *Caulobacter* *crescentus* NA1000 (accession number NC_011916), *E. coli* K-12 MG1655 (accession number NC_000913). 0.43% of *Vibrio* genomes (61) have genes for the complete xylose isomerase pathway while 10.04 % of *E. coli* species have the genes. None of the two stains has the whole pathways for the oxidoreductase pathway, the Weimberg pathway, or Dahms pathway. A number in parenthesis indicates the number of strains having a xylose pathway. ND, Not detected.

## Figure S2. Known xylose catabolic pathways


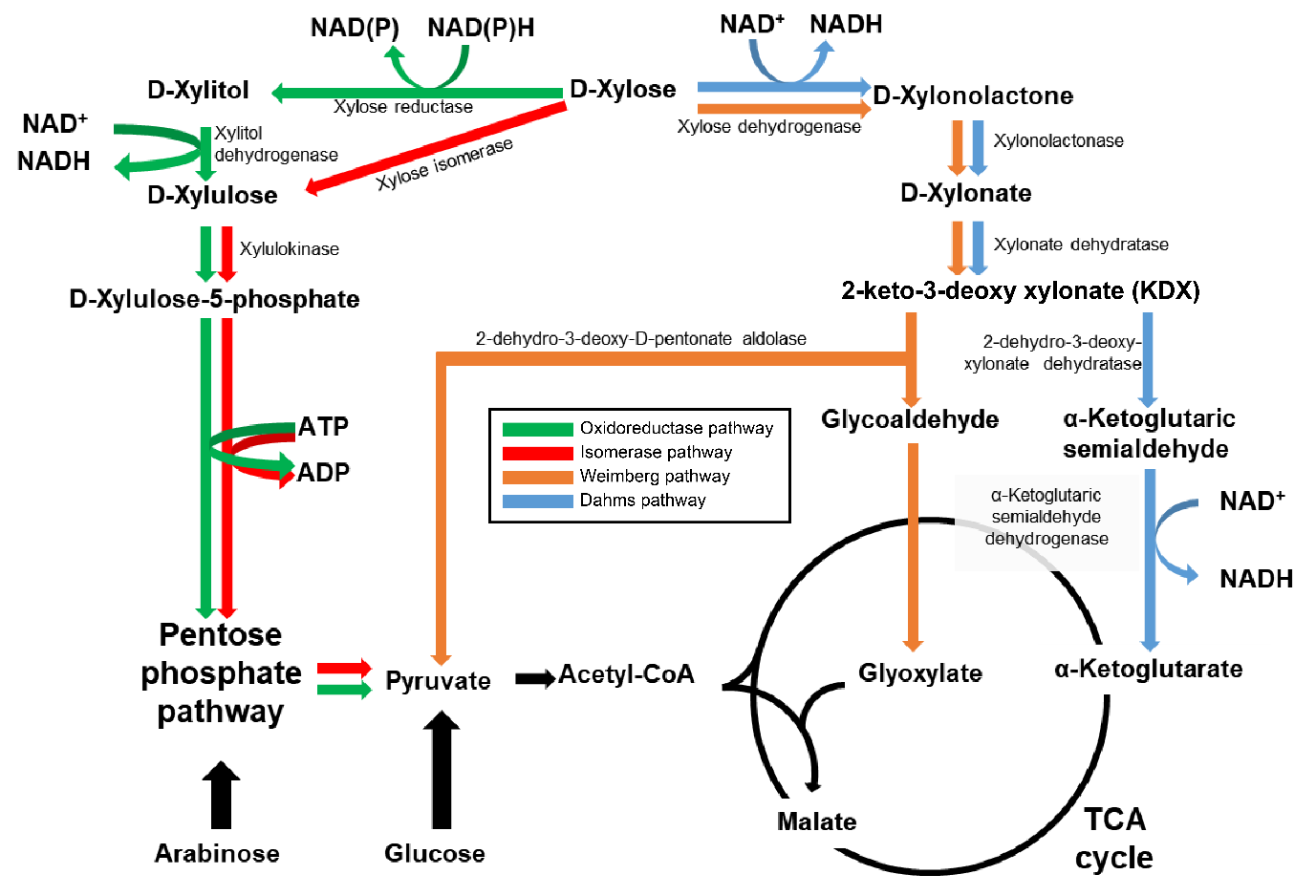


The xylose isomerase pathway (red arrows), oxidoreductase pathway (green arrows), Weimberg pathway (blue arrows), Dahms pathway (orange arrows) were shown. The xylose isomerase pathway is common in bacteria while the oxidoreductase pathway was reported in several yeast strains. With these pathways, xylose is metabolized through the pentose phosphate pathway (PPP) after isomerization into xylulose. The difference between the two pathways is that the isomerase pathway uses isomerase to convert xylose into xylulose, whereas xylose is reduced to xylitol, and then it is re-oxidized to xylulose in the oxidoreductase pathway. In some bacteria and archaea [14, 15], xylose is catabolized by the Weimberg pathway or Dahms pathway. With the two pathways, xylose is oxidized and dehydrated to 2-keto-3-deoxy xylonate (KDX). In the Weimberg pathway, KDX is dehydrated and then oxidized to alpha-ketoglutarate. In the Dahms pathway, KDX is split into pyruvate and glycolaldehyde by aldolase.

## Figure S3. Growth profiles of the VXA1-1, VXA1-2, VXA15-1, and VXA15-3 strains in xylose minimal medium


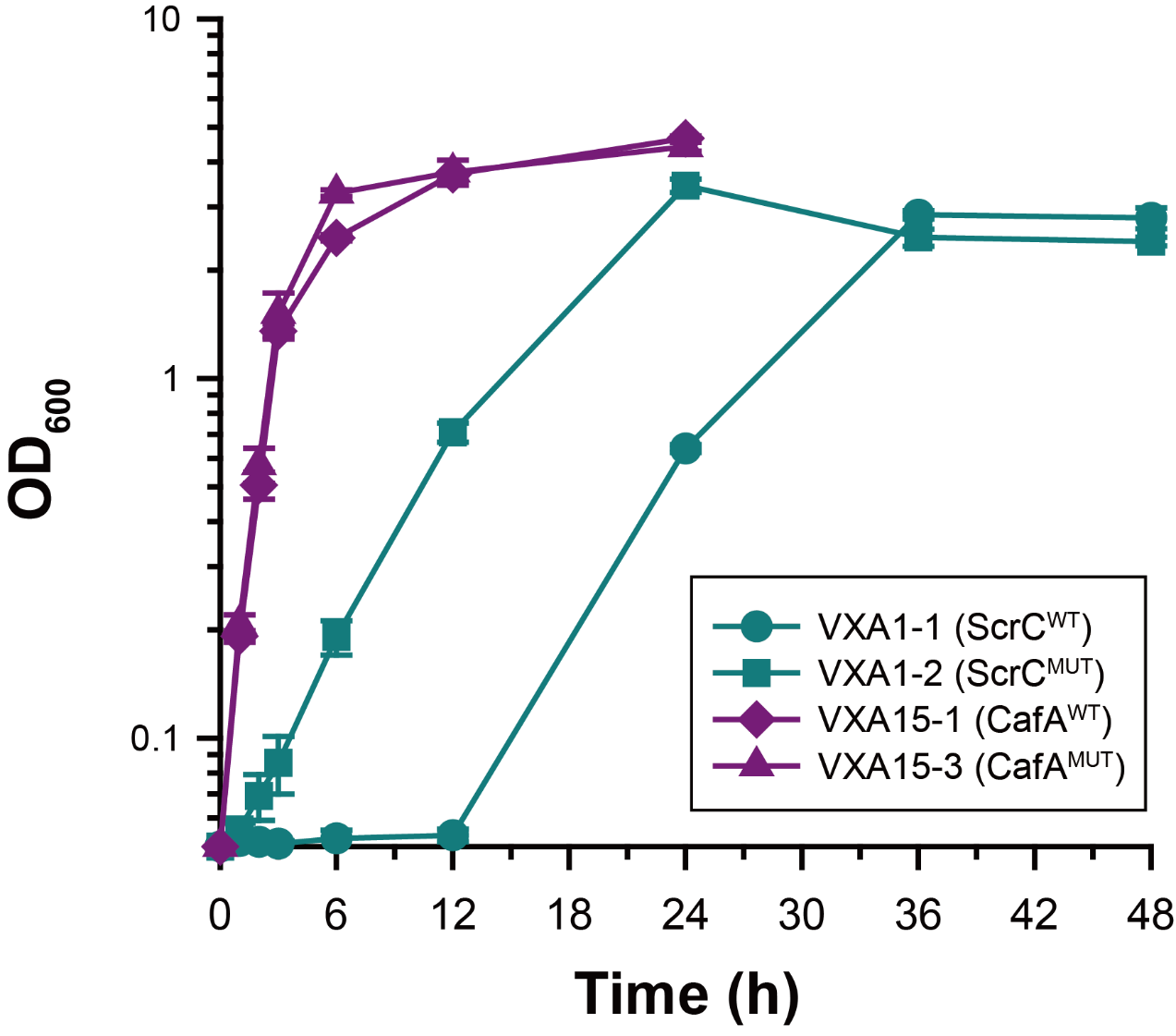


Cell growth (OD_600_) of the VXA1-1, VXA1-2, VXA15-1, and VXA15-3 strains in the xylose minimal medium. Symbols: cyan circle, VXA1-1 (expressing wild-type ScrC); cyan square, VXA1-2 (expressing mutant ScrC); magenta diamond, VXA15-1 (expressing wild-type CafA); magenta triangle, VXA15-3 (expressing mutant CafA)

## Figure S4. Sequence alignment of xylulokinase (XylB) from *E. coli* and *Vibrio* sp. dhg


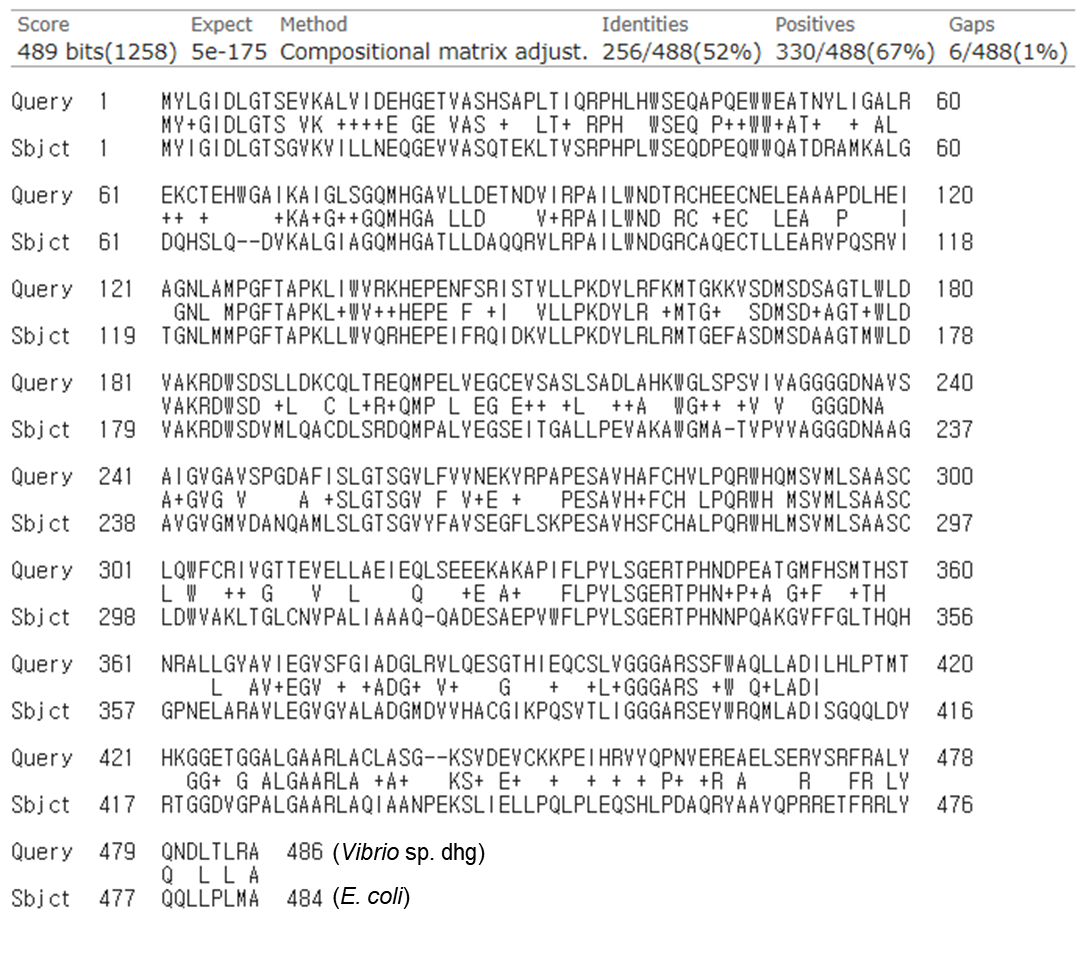


Query sequence and subject sequence are the amino acid sequences of *Vibrio* sp. dhg XylB (485 amino acids, NCBI accession number: AXT73989.1) and *E. coli* XylB (484 amino acid), respectively. BLAST (https://blast.ncbi.nlm.nih.gov/Blast.cgi) was used for the sequence alignment (score, 489; query cover, 100%; E-value, 5e-175; percentage identity, 52.46%).

## Figure S5. Comparison of K_m_ and *k*_cat_ of the wild-type and mutant xylose isomerase


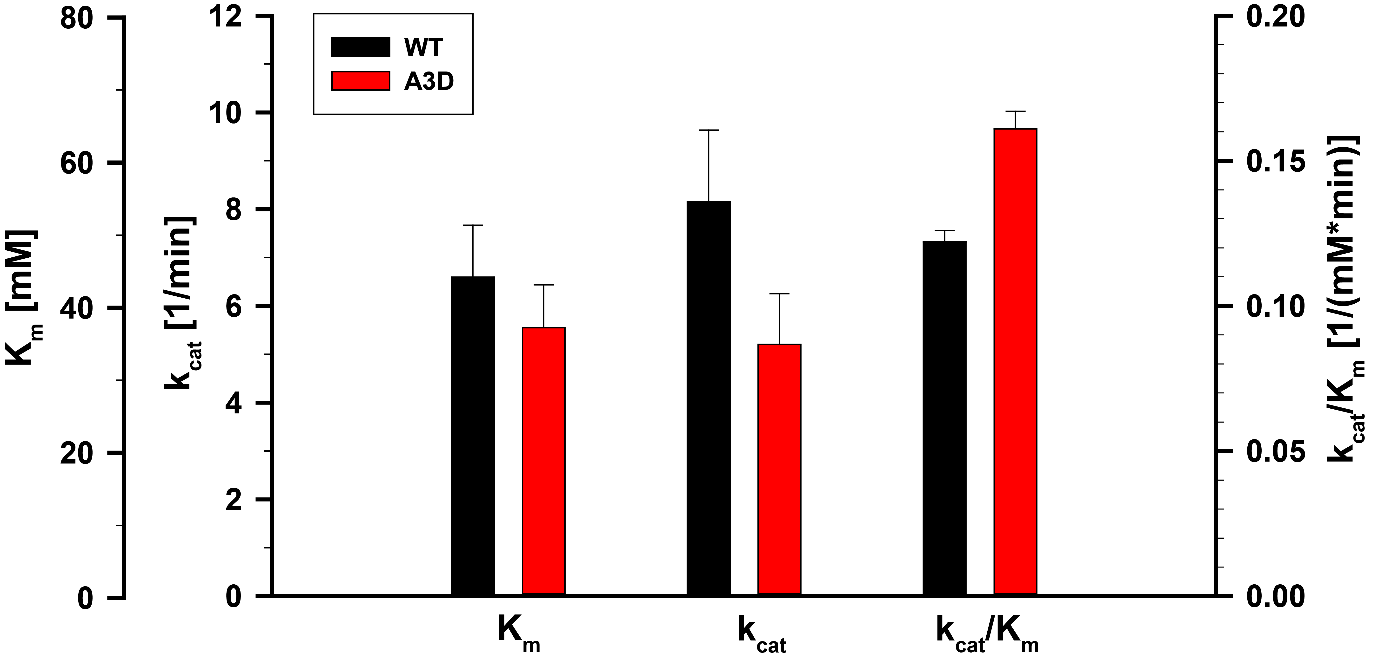


K_m_ and *k*_cat_ of 6His-tagged wild-type (black bar) and mutant (red bar) XylA via enzymatic assay. K_m_ and *k*_cat_ were calculated through the Lineweaver-Burk equation [16]. K_m_ and *k*_cat_ were 0.64-fold and 0.83-fold decreased, respectively, resulting in a 1.3-fold higher *k*_cat_/K_m_.

## Figure S6. Normalized specific fluorescence values of strains expressing the *xylA-sgfp* fused protein with wild-type and mutant promoter and *xylA* coding sequence.


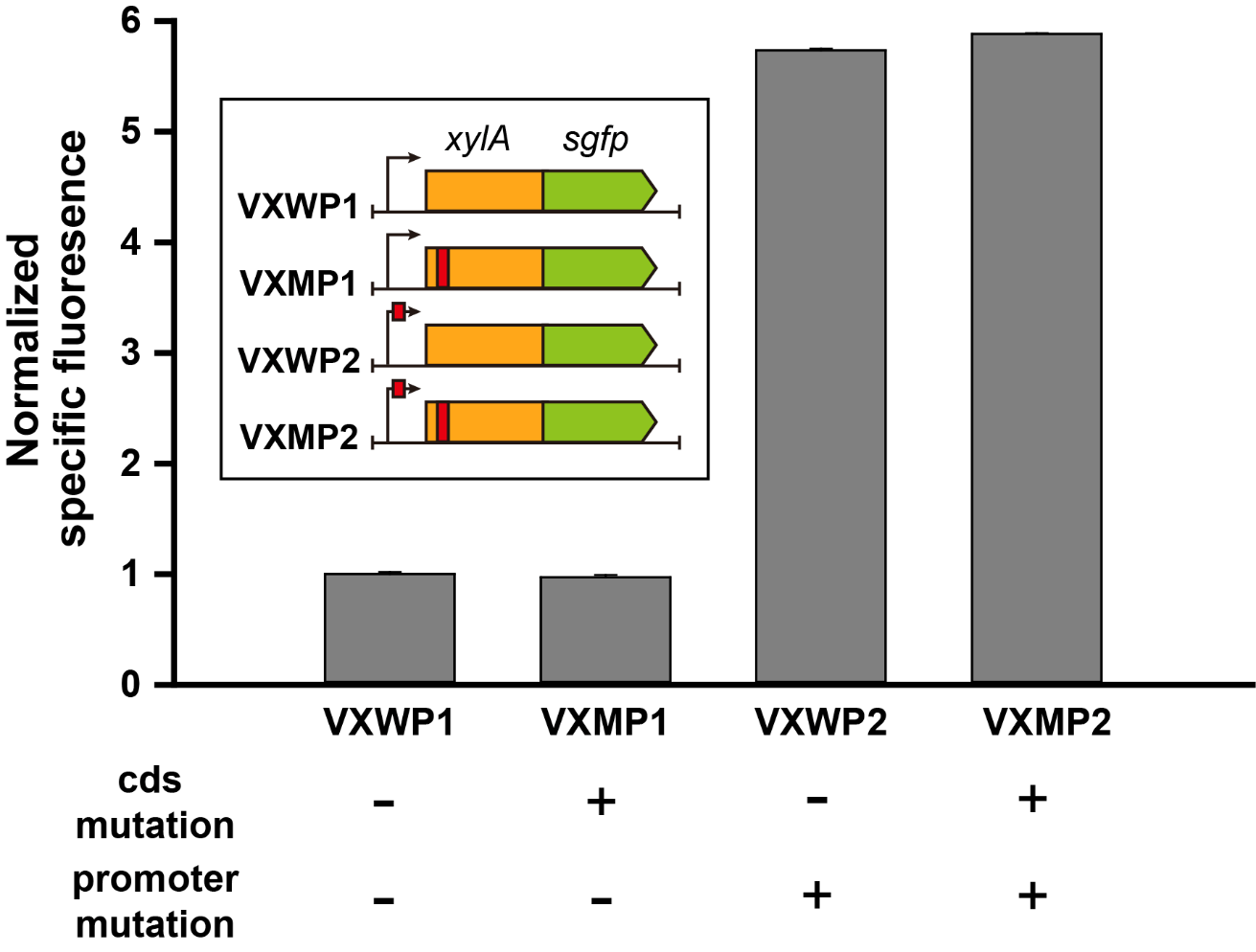


Changes in expression level according to the mutations on the promoter (-35C>A) and coding sequence (A3D) were investigated by measuring specific fluorescence of *xylA-sgfp* fused protein. Although promoter mutation increased expression level by 6-fold, mutation on coding sequence did not affect expression level. Each specific fluorescence was normalized by the value of that of VXWP1 strain as a control.

# Reference

1. Skoog K, Hahn-Hägerdal B. Effect of oxygenation on xylose fermentation by *Pichia stipitis.* *Appl. Environ. Microbiol*. 1990;56:3389–3394. doi:10.1128/AEM.56.11.3389-3394.1990.

2. Liu X, Yang S-T. Kinetics of butyric acid fermentation of glucose and xylose by *Clostridium tyrobutyricum* wild type and mutant. *Process Biochem.* 2006;41:801–808. doi:10.1016/j.procbio.2005.10.009.

3. Radek A, Krumbach K, Gätgens J, Wendisch VF, Wiechert W, Bott M, et al. Engineering of *Corynebacterium glutamicum* for minimized carbon loss during utilization of D-xylose containing substrates. *J. Biotechnol*. 2014;192 Pt A:156–160. doi:10.1016/j.jbiotec.2014.09.026.

4. Peng B, Huang S, Liu T, Geng A. Bacterial xylose isomerases from the mammal gut *Bacteroidetes* cluster function in *Saccharomyces cerevisiae* for effective xylose fermentation. *Microb. Cell Fact.* 2015;14:70. doi:10.1186/s12934-015-0253-1.

5. Bator I, Wittgens A, Rosenau F, Tiso T, Blank LM. Comparison of three xylose pathways in *Pseudomonas putida* KT2440 for the synthesis of valuable products. *Front. Bioeng. Biotechnol.* 2019;7:480. doi:10.3389/fbioe.2019.00480.

6. Hernández-Montalvo V, Valle F, Bolivar F, Gosset G. Characterization of sugar mixtures utilization by an *Escherichia coli* mutant devoid of the phosphotransferase system. *Appl. Microbiol. Biotechnol*. 2001;57:186–191. doi:10.1007/s002530100752.

7. Den Dunnen JT, Dalgleish R, Maglott DR, Hart RK, Greenblatt MS, McGowan-Jordan J, et al. HGVS recommendations for the description of sequence variants: 2016 update. *Hum. Mutat.* 2016;37:564–569. doi:10.1002/humu.22981.

8. Yoshida S, Okano K, Tanaka T, Ogino C, Kondo A. Homo-D-lactic acid production from mixed sugars using xylose-assimilating operon-integrated *Lactobacillus plantarum*. Appl. *Microbiol. Biotechnol*. 2011;92:67–76. doi:10.1007/s00253-011-3356-6.

9. Tanaka K, Komiyama A, Sonomoto K, Ishizaki A, Hall SJ, Stanbury PF. Two different pathways for D-xylose metabolism and the effect of xylose concentration on the yield coefficient of L-lactate in mixed-acid fermentation by the lactic acid bacterium *Lactococcus lactis* IO-1. *Appl. Microbiol. Biotechnol.* 2002;60:160–167. doi:10.1007/s00253-002-1078-5.

10. Zhao J, Xu L, Wang Y, Zhao X, Wang J, Garza E, et al. Homofermentative production of optically pure L-lactic acid from xylose by genetically engineered *Escherichia coli* B. *Microb. Cell Fact.* 2013;12:57. doi:10.1186/1475-2859-12-57.

11. Dien BS, Nichols NN, Bothast RJ. Fermentation of sugar mixtures using *Escherichia coli* catabolite repression mutants engineered for production of L-lactic acid. *J. Ind. Microbiol. Biotechnol*. 2002;29:221–227. doi:10.1038/sj.jim.7000299.

12. Lu H, Zhao X, Wang Y, Ding X, Wang J, Garza E, et al. Enhancement of D-lactic acid production from a mixed glucose and xylose substrate by the *Escherichia coli* strain JH15 devoid of the glucose effect. *BMC Biotechnol*. 2016;16:19. doi:10.1186/s12896-016-0248-y.

13. Lim HG, Kwak DH, Park S, Woo S, Yang JS, Kang CW, et al. *Vibrio* sp. dhg as a platform for the biorefinery of brown macroalgae. *Nat. Commun*. 2019;10:2486. doi:10.1038/s41467-019-10371-1.

14. Dahms AS. 3-Deoxy-D-pentulosonic acid aldolase and its role in a new pathway of D-xylose degradation. *Biochem. Biophys. Res. Commun.* 1974;60:1433–1439. doi:10.1016/0006-291X(74)90358-1.

15. Weimberg R. Pentose oxidation by *Pseudomonas fragi*. *J. Biol. Chem.* 1961;236:629–635.

16. Lineweaver H, Burk D. The determination of enzyme dissociation constants. *J. Am. Chem. Soc*. 1934;56:658–666. doi:10.1021/ja01318a036.
